# Functional hemispheric asymmetries during the planning and manual control of virtual avatar movements

**DOI:** 10.1371/journal.pone.0185152

**Published:** 2017-09-28

**Authors:** Mareike Floegel, Christian Alexander Kell

**Affiliations:** Cognitive Neuroscience Group- Brain Imaging Center and Department of Neurology, Goethe University, Frankfurt, Germany; University of Zurich, SWITZERLAND

## Abstract

Both hemispheres contribute to motor control beyond the innervation of the contralateral alpha motoneurons. The left hemisphere has been associated with higher-order aspects of motor control like sequencing and temporal processing, the right hemisphere with the transformation of visual information to guide movements in space. In the visuomotor context, empirical evidence regarding the latter has been limited though the right hemisphere’s specialization for visuospatial processing is well-documented in perceptual tasks. This study operationalized temporal and spatial processing demands during visuomotor processing and investigated hemispheric asymmetries in neural activation during the unimanual control of a visual cursor by grip force. Functional asymmetries were investigated separately for visuomotor planning and online control during functional magnetic resonance imaging in 19 young, healthy, right-handed participants. The expected cursor movement was coded with different visual trajectories. During planning when spatial processing demands predominated, activity was right-lateralized in a hand-independent manner in the inferior temporal lobe, occipito-parietal border, and ventral premotor cortex. When temporal processing demands overweighed spatial demands, BOLD responses during planning were left-lateralized in the temporo-parietal junction. During online control of the cursor, right lateralization was not observed. Instead, left lateralization occurred in the intraparietal sulcus. Our results identify movement phase and spatiotemporal demands as important determinants of dynamic hemispheric asymmetries during visuomotor processing. We suggest that, within a bilateral visuomotor network, the right hemisphere exhibits a processing preference for planning global spatial movement features whereas the left hemisphere preferentially times local features of visual movement trajectories and adjusts movement online.

## Introduction

Many daily goal-directed actions, especially performed by the hands, occur in a visual context. Vision provides spatial information about the location, size, and shape of objects as well as one’s own limbs (visuospatial component). Further, vision carries information about the time point or order of visual events (visuotemporal component). This information can be used to acquire internal action representations and to refine and update actions once a movement is learned [1–4]. When playing computer games or navigating the computer cursor to a specific location on a screen, a virtual avatar is easily controlled by the force that is applied to the controller like a mouse, joystick, or touchpad. In this context, the actual hand action (e.g. isometric force on a device) is coupled to the movement of a virtual avatar in space. Vision provides information about the current state of the visual avatar and the visual consequences of the hand action, which allows differences between the current and desired state of the virtual avatar by means of action to be minimized [5]. Hereby, hand motion is not necessarily required and can be replaced by other actions (e.g. isometric grip force) as long as the relationship between the visually perceived virtual avatar movement and action is predictable [6]. The ease with which this happens suggests that, indeed, visual input can easily guide action, also when proprioceptive feedback from the hand does not map directly onto the visual movement. So far, it is unclear how neural resources during visuomotor processing, i.e. the transfer of visual information into action [7], are distributed over the two hemispheres. A consistent finding in both visual perception and in motor control studies is the functional asymmetry between the two halves of the brain. Strikingly, researchers working in perception propose very different origins of hemispheric specialization than researchers investigating motor control. Yet, perceptual and motor systems are heavily intertwined.

Work in motor control suggests the left hemisphere controls movement sequencing [8, 9], temporal processing [10], response selection [11] or tool use and prehension [12, 13]. Accordingly, up to 90% of the human population perform motor tasks better with their right than left hand [14] and sequential complex or rapid movements are slower and more inaccurate in left (but not right) hemisphere-damaged patients relative to controls [8, 15]. In the field of motor control, the right hemisphere is often thought to contribute sensory information [16, 17] compared to the more output-related processing in the left hemisphere [17].

Yet, research in perception has provided a more refined picture in which also the left hemisphere contributes to perception and provides information that could be used for producing purposeful movements. From a purely perceptual context it is known that the right hemisphere is important for most visuospatial tasks [18–22], whereas the left hemisphere is more involved in temporal evaluation of visual stimuli [23–24]. Thus, functional hemispheric asymmetries underlying perception may be driven by preferential visuospatial and visuotemporal processing in the right and left hemispheres, respectively. A more general framework about hemispheric specialization in perception posits that the observed right hemisphere’s specialization for processing of spatial information is not absolute but rather attributable to frequency-dependent filtering of sensory information in the two hemispheres (Double filtering by frequency theory, DFF [25]). The right hemisphere has been proposed to preferentially decode low visuospatial frequency information leading to a rather global representation of the visual scene. This adds weight to the overall spatial relationship between visual elements. Conversely, the left hemisphere is thought to preferentially decode high visuospatial frequency information leading to a more detailed, but less integrated representation of the visual scene [25] which allows fine, local perceptual discriminations. The left hemisphere processing preference for local stimulus features and right hemisphere preference for global features is also observed in non-human primates [26, 27].

Behavioral data obtained from split-brain patients suggest that, compared to perception, the degree of functional lateralization may even be higher if visual input specifies manual actions [28]. This suggests that hemispheric asymmetries may be stronger for visuomotor processing compared to visual perception alone. While right-lateralization of visuospatial processing is well-documented for purely *perceptual* tasks, such an effect has not been consistently found for visuospatial processing during visuo*motor* tasks [for examples of visuomotor processing and right lateralization see, [16], and for reports on visuomotor processing and left lateralization, see [29, 30]].

Thus, functional lateralization of visuomotor processing is not clear. Two alternative hypotheses can be formulated. Input-related sensory processing is lateralized to the right hemisphere and output-related processing is left-lateralized (see above). Alternatively, the two hemispheres contribute differently to input-related sensory processing during visuomotor tasks. Specifically, we hypothesized that global spatial processing of the sensory input lateralizes to the right, while local spatial processing and temporal processing of the sensory input lateralizes to the left hemisphere during visuomotor processing (see above). Given that lateralization discrepancies in previous studies may be attributed to different spatiotemporal processing demands during visuomotor planning or online visuomotor processing [31] the present study explicitly considered planning- and online processing-related effects. Investigating the control of a virtual avatar by grip force, which, in comparison to reaching and grasping, does not entail a hand and arm movement in visual and proprioceptive space, reduced effector-dependence of lateralization effects.

Functional imaging studies have shown that the visuomotor network to control the visual movement of a virtual avatar by means of isometric grip force comprises bilateral visual cortices, premotor areas (dorsal premotor cortex (PMd) and ventral premotor cortex (PMv)), supplementary motor area (SMA), cingulate motor area (CMA), the inferior parietal lobe (IPL), superior parietal lobe (SPL), intraparietal sulcus (IPS), the insula, rolandic operculum, and subcortical areas like the thalamus, putamen, and cerebellum [32]. Further, the network includes primary motor (M1) and somatosensory (S1) cortices contralateral to the hand used [33]. Studies investigating prehensive movements indicate that activity and lateralization in this network may be modulated by the hand used to perform the action [34] and handedness [34, 35], though effects of these two factors on behavior are rather small [36, 37]. Yet, functional lateralization in the aforementioned network and its potential modulation by sensory input has not been systematically investigated. Given the increasing importance of virtual avatars in a more and more device-oriented environment, a better understanding of neural processing during visuomotor processing is important.

Nineteen right-handed healthy participants regulated isometrically applied grip force on a manipulandum to control the visually perceived spatial position of a movable cursor on a reference trajectory [7, 38–41]. An increase in grip force produced an upward movement of the cursor. The reference trajectories differed between three experimental conditions. They indicated with varying degree *when* the cursor should move (*temporal control demands*) and where the curser should move (*spatial control demands*) and therewith when and how strong force should be applied. Particularly, the trajectories prompted either a continuous up movement of the cursor up to a defined position (*high spatial*, *high temporal processing demands*), a quasi stationary cursor position above the home position (*high spatial*, *low temporal processing demands*), or the timing of several short ballistic upward cursor movements without spatial specifications (*low spatial*, *high temporal processing demands*). We tested hand-specific functional asymmetries but more importantly which brain areas displayed higher BOLD responses in one hemisphere in comparison with the other, independent of whether the right or left hand performed the task. BOLD signal asymmetries were separately investigated during planning of and online-control of visually guided cursor movements. Hereby, spatial visuomotor planning refers to processes that translate the required spatial position change of the curser into isometric grip force. Temporal visuomotor planning refers to processes that time the production of grip force according to visual information. Specifically, we hypothesized that visuospatial information is processed more globally (lower relative spatial frequency) for planning virtual avatar movements, whereas visuospatial information is processed more locally (higher relative spatial frequency) for fine spatial and temporal adjustments of the ongoing virtual avatar movement (detection of small deviations between visual perceived actual and desired virtual avatar position).

## Methods

### Participants

20 healthy volunteers (10 female) with normal or corrected to normal vision participated in the study. All participants were free of magnetic resonance imaging (MRI) contraindications and gave their written informed consent before participation. One male participant was excluded from further analysis, since the 10 Item version of the Edinburgh handedness inventory [42] indicated strong left-handedness (score = -100). The remaining 19 participants (Age: mean = 25 years, SD = 2.77, range 22–30) were right-handed (mean score = 88.42, SD = 16.75). The study was approved by the ethics committee of the Medical Faculty of Goethe-University Frankfurt and was in accordance with the Declaration of Helsinki.

### Virtual avatar control

During the experiment, participants performed two successive sessions during which they controlled a virtual avatar by grip force. Participants operated two cursors along reference trajectories by increasing and decreasing the exerted force on two MR-compatible hand dynamometers (TSD121B-MRI, BIOPAC Systems, Inc.; Goleta, CA; USA). Throughout the experiment, one dynamometer was held in the right and the other in the left hand with a power grip, i.e. with the thumb opposing the other four fingers. On each trial, only one of the two dynamometers had to be used (unimanual conditions). The order of conditions and hands to be used was randomized. Participants lay supine in the scanner and saw a visual display (see below) with the cursors and reference trajectories through a mirror attached to the headcoil. The upper-arms rested beside the body. Participants were instructed to keep the cursors on the reference trajectories but to remain otherwise motionless. Before scanning, but already inside the scanner, participants familiarized themselves with the sensitivity of the hand dynamometers and practised the task. The sensitivity of the dynamometers was scaled according to the individual participant’s maximal voluntary contraction (MVC). MVC was defined as the mean force value when squeezing the dynamometer for 2s as strongly as possible. The maximum required force output of both hands during the task was standardized to 10% MVC of the right hand.

### Visual display

**Fig 1:**
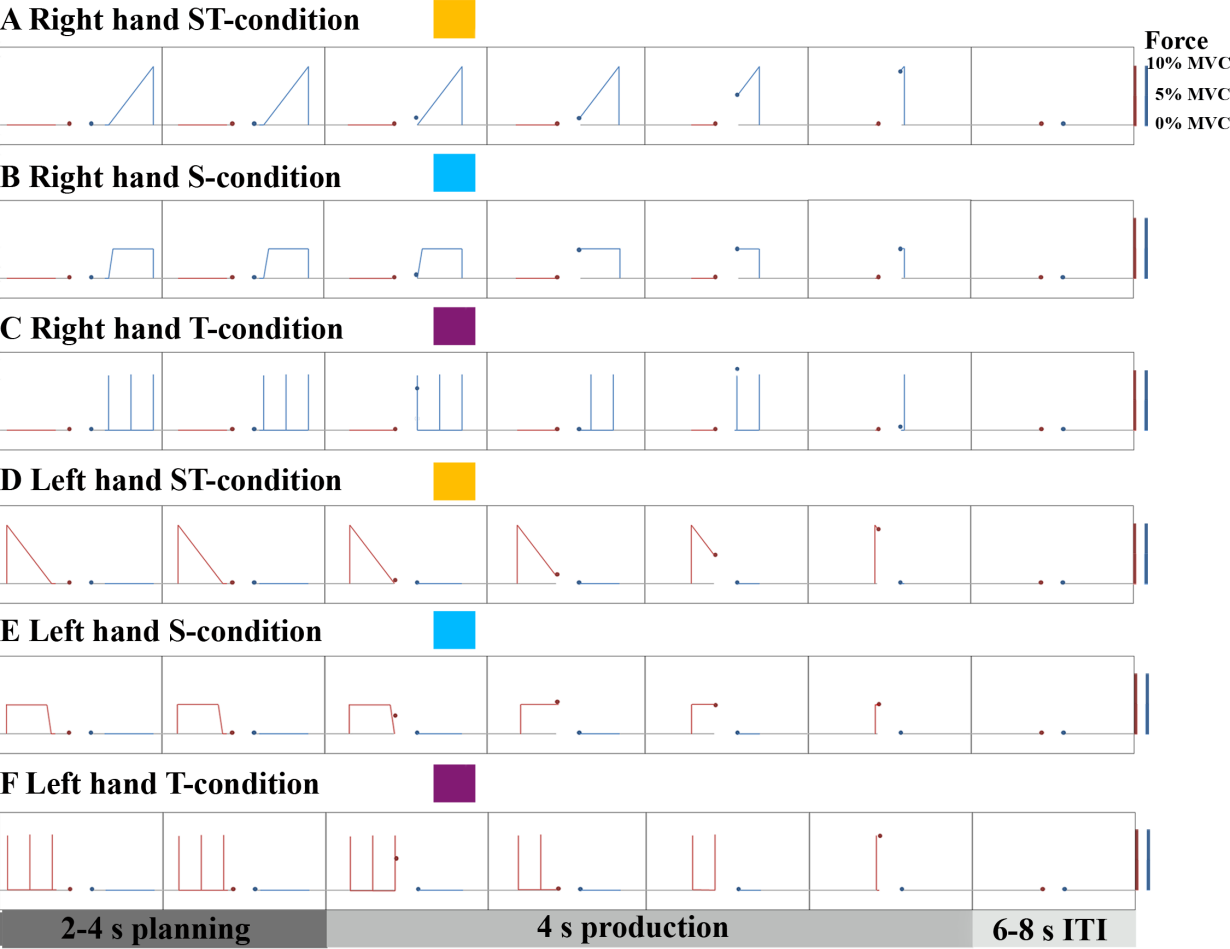
Experimental conditions and trial structure. Time is illustrated from left to right. Each trial started with a 2-4s long planning phase during which the cursors were in the vertical home position (left cursor: red point, right cursor: blue point). The inward moving red or blue lines indicated the reference trajectories that could be planned until the start of the colored line reached the vertical plane of the cursors. This served as a Go Signal for the 4s production phase. The Intertrial Interval (ITI) was jittered between 6-8s. Increasing force (illustrated as % of maximal voluntary contraction, MVC) resulted in a vertical deflection of the cursors. The trajectories of the three different conditions are exemplified for the right hand (A-C) and left hand (D-F). (A/D) Trajectory of the condition with high spatial and temporal processing demands (ST-condition), (B/E) with high spatial and low temporal processing demands (S-condition), or (C/F) with low spatial and high temporal processing demands (T-condition). The color codes that are indicated next to the description of the conditions are used throughout the manuscript to illustrate condition effects.

The visual display (Fig 1 and S1 Video) consisted of two vertically movable cursors in the middle of a screen and two inward moving lines (one from left to right, the other from right to left, 6 cm/s). Force on the right dynamometer changed the vertical position of the right cursor (blue) and force on the left dynamometer changed the vertical position of the left cursor (red) in a linear fashion (increase in force resulted in upward movement of the cursor). The cursor could not be moved horizontally. The vertical deflection of the inward moving lines compared to the horizontal axis representing the home position (0% MVC) represented the instructed cursor position.

Participants were instructed to operate the right and left cursor so that the actual vertical deflection of the cursor matched the vertical deflection of the reference (indicated by the inward moving lines). The go signal was defined as the moment the inward moving lines touched the vertical planes of the cursors. Participants were instructed to fixate the middle of the screen marked by a grey circle. Accordingly, horizontal saccades should have been kept at a minimum. However, eye movements were not explicitly controlled for in the scanner. Peripheral vision allowed for planning visuospatial and visuotemporal cursor movement features from the moment the lines appeared in the periphery until the go signal (planning phase). Specifically, the inward movement of the reference trajectory from the periphery to the center of the visual display allowed viewing the course of expected positions (i.e. the expected cursor movement) even before the lines touched the vertical planes of the cursor.

Spatiotemporal properties of the reference trajectories varied systematically. Because we wanted to investigate spatial and temporal aspects of visuomotor processing, we studied three different, unimanual conditions during which either spatial or temporal demands predominated or during which these factors were expected to be equally relevant. Each condition was performed 10 times with the left hand and 10 times with the right hand. The *high spatial*, *high temporal processing demands (ST)* condition consisted of a continuous increase in expected cursor position resulting in a linear upward movement of the cursor (Fig 1A and 1D). The condition required an increase of hand grip force over 4 seconds until 10% MVC was reached. During the *high spatial*, *low temporal processing demands (S)* condition the expected cursor position was steady at a vertical deflection that corresponded to 5% MVC for 4 seconds (Fig 1B and 1E). Thus, participants had to keep the cursors quasi stationary in the middle of the screen. The mean force of both conditions with high spatial processing demands (ST and S) was 5% MVC for better inter-condition comparison. Finally, the *low spatial*, *high temporal processing demands (T)* condition instructed a deflection from the home position at discrete time points (Fig 1C and 1F). The extent of cursor position change (spatial feature) was not controlled. This condition resulted in three ballistic upward movements of the cursor.

### Trial phases

Each trial started with a planning period in which the upcoming trajectory was already seen but in which action was not yet required. This planning phase was jittered between 2–4 seconds [steps of 0.5 s mean: 3s] to reduce temporal correlation between processing phases and to delineate the hemodynamic response during visuomotor planning from the subsequent response during the actual cursor movement. The movement period was chosen to be 4 seconds since this duration enabled detection of task-related brain activity in a previous event-related fMRI study [43]. The inter-trial interval was on average 7s (jittered between 6-8s). In total, participants performed each condition 10 times with each hand within a session. Conditions were presented in randomized order. Overall, 20 trials per condition were collected per participant resulting in a total scanning time of 40 minutes.

### Data acquisition

Applied grip force was measured with MR-compatible hand clench dynamometers (TSD121B-MRI) connected to the BIOPAC MP150 acquisition system (BIOPAC Systems, Inc.; Goleta, CA, USA) via the corresponding cable/filter system. The measured grip strength (up to 50 kg) was directly obtained in kilograms and recorded with the software AcqKnowledge 4.3.1 (BIOPAC Systems, Inc.; Goleta, CA, USA). Force was sampled at 1000 Hz. Custom made software translated force values online in the virtual avatar movement. Latency of displaying information on the screen (< 40 ms) was measured by counting the number of drawn frames during a run and dividing this number by a run’s length. Perceptually, no participant reported experiencing a response delay.

Scanning was performed using a Siemens (Erlangen, Germany) Trio 3 Tesla magnetic resonance scanner with a CP Send/Receive head coil. Functional images were obtained with a gradient-echo T_2_*-weighted transverse echoplanar image (EPI) sequence [603 volumes per run (in total 1206 volumes); TR = 2s; TE = 30ms; flip angle = 90°; 33 axial slices; 3mm^3^ isotropic voxel size]. In addition, high-resolution T_1_-weighted anatomical scans [TR = 2.25s; TE = 3.83ms; flip angle = 9°; 144 slices per volume; 1mm^3^ isotropic voxel size] were obtained to improve spatial normalization of functional images onto the Montreal Neurological Institute (MNI) brain template.

### Data analysis

#### Behavioral data

For each participant and trial, the performed trajectory of the virtual avatar (i.e. the cursors) was plotted against the expected trajectory to identify missing or incorrect trials by means of visual inspection. A trial was labeled as incorrect if movement was already observed during the planning period (in total 2 trials), if the wrong cursor was operated (i.e. wrong hand exerted force, in total 2 trials) or if the trajectory was not correctly traced (in total 5 trials).

For the correct trials, mean force and trial to trial variability, the latter used as a measure of performance stability, were calculated separately for each condition according to formula 1–3 below in which *t* denotes the number of trials and *n* the number of sampling points during the virtual avatar movement. To test whether there were any differences between conditions at the behavioral level, mean force and trial to trial variability were entered into two two-factorial repeated measures ANOVAs testing main effects of condition and hand as well as their interaction (*p* < 0.05). Despite training prior to the main experiment we checked whether participants increased or decreased their performance throughout the experiment. We compared the mean force deviation of the first 10 trials within a condition with the mean force deviation of the last 10 trials of that same condition (p < 0.05, Bonferroni corrected).

$$mean\;force=\frac{1}{t}\sum_{l=1}^{t}\frac{\sum_{i=1}^{n}{actual\;force}_{li}}{n_{l}}$$ (1)

$${mean\;force\;deviation}_{over\;all\;trials}=\frac{1}{t}\sum_{l=1}^{t}\frac{\sum_{i=1}^{n}{|actual\;force}_{li}-{expected\;force}_{li}|}{n_{l}}$$ (2)

$$trial\;to\;trial\;variability=\frac{\sum_{l=1}^{t}({mean\;force\;deviation}_{i}-{mean\;force\;deviation}_{over\;all\;trials}{)}^{2}}{t-1}$$ (3)

#### fMRI data

Image processing and statistical analysis was performed using SPM12 (Wellcome Trust Centre for Neuroimaging, London, UK; http://www.fil.ion.ucl.ac.uk/spm). The spatial preprocessing procedure used standard SPM 12 parameters and encompassed the following steps: 1) Realignment of functional images using rigid body transformation, 2) co-registration of subject’s individual structural scans with the mean functional image of the realignment step, 3) normalization of functional images to the Montreal Neurological Institute (MNI) standard brain template within the Talairach and Tournoux reference frame via parameters from the segmentation procedure of structural scans and 4) smoothing of images with an isotropic 8 mm full-width at half-maximum (FWHM) Gaussian kernel. Afterwards, the preprocessed functional images were analyzed within the framework of general linear models adapted for non-spherical distributed error terms [44] as implemented in SPM 12.

### Model specification

The present study considered lateralized effects for visuomotor planning and online visuomotor processing separately. The standard SPM modeling approach comes at the cost of considerable temporal correlations between the regressors modelling the temporally close and non-permutable events of visuomotor planning and online visuomotor processing (in our data: r = 0.52). Temporal correlations between regressors could have been reduced by longer jitter periods, but this was unsuitable in the present case. From a psychological perspective, participants would have likely started preparing their movements not right from the beginning of the planning phase but somewhen at the end, if the temporal jitter was too large. Residual correlations between regressors do not just lead to underestimated beta estimates with high variances [45, 46] but may also affect the sign of beta weight estimates [47–49]. Thus, negative beta weights may be observed (negative suppression or net suppression) even though all variables are correlated positively with each other. This renders the interpretation of effects in a model comprising both processing phases impossible.

For this reason, three reduced general linear models were estimated and compared for each participant to decide, for each voxel separately, whether its BOLD response was overall better predicted by a model assuming activation only during visuomotor planning, only during online visuomotor processing or throughout both trial phases [50]. Each model comprised both sessions of the virtual avatar control task, high pass filtered with a high pass cut off at 128s to remove low frequency drifts, and an autoregressive model AR(1) to account for serial autocorrelations in the time series. The three models either consisted of regressors timed to planning- (model 1) or online processing-related (model 2) events or combined planning- and online processing-related events in a single regressor (model 3). Planning-related regressors were defined by the start of a trial where the cursor’s movement trajectory was already seen, but force production not yet required. Duration was defined as the interval between trial onset and measurable cursor position change (force > 0.05% MVC). Online processing-related regressors were specified according to the time point when participants actually altered the cursor position. Their duration was specified as the interval between start and end of measurable force exertion. The onset of combined regressors reflecting planning and online processing were specified according to the start of a trial and lasted until the end of measurable force. The three models were voxel-wise compared to reduce the risk of misattributing activation to a movement phase that contributed only little to the observed blood oxygen level dependent (BOLD) time-course. For each voxel and participant, we assessed whether log residual variance (ImCalc function of SPM) was smallest (greater model fit, more explained variance in the BOLD signal time-course) for the planning, online processing or combined model [50]. Afterwards, voxels whose BOLD signal changes were better described by the planning relative to the online processing and combined model (Planning_log(residualVariance)_ < Execution _log(residualVariance)_ ∩ Planning _log(residualVariance)_ < Control _log(residualVariance)_, *p* < 0.05, uncorrected) were collected in a mask that was used for subsequent statistical interference in the planning model. The same was done for voxels whose BOLD response was better characterized by the online processing model or combined model. Of note, the relative superiority of one model relative to the other does not allow one to decide whether a brain region’s activity represents exclusive processing related to visuomotor planning or online processing or is very similar between both movement phases. However, it suggests whether the relative contribution of planning and online processing to the BOLD response in a region likely differs or not.

The design matrices of the three models each consisted of 6 regressors of interest, modelling the three different conditions with either the left or the right hand, and 12 regressors of no interest, obtained from the realignment step, modelling movement-related effects (2x3 rotations and translations). In all three models, condition-specific regressors were obtained by convoluting the onset and duration of conditions (modelled by boxcar functions) with the canonical hemodynamic response function (HRF). Missing trials or trials with a wrong response were not explicitly modelled due to their infrequent occurrence and entered into silent baseline.

### Statistical inference

#### Network of visuomotor planning and online visuomotor processing

After estimation of the models, contrasts were specified testing the effect of each regressor of interest against baseline in each individual (first-level). To allow inferring brain activation at the population level, the resulting contrast images of each participant were subjected to a second level random effect (RFX) analysis. For each model a flexible factorial 2x3 ANOVA was used that allowed the inclusion of subject effects [51] and interaction terms to investigate dependencies between factors. The ANOVA modelled the main effect of the factor hand (left and right hand), the main effect of the factor condition with three factor levels (ST, S, T), and the interaction of both factors (6 regressors). Please note that the main effect of hand and other hand-specific observations may also have been driven by visual-field effects. This could not be excluded since right cursor movements were always instructed by the right line and performed with the right hand and left cursor movements were always instructed with the left line and performed with the left hand. For simplification, these effects will only be referred to as hand-specific.

A conjunction analysis testing the conjunction null hypothesis [52] was performed to identify the common network for visuomotor planning and online visuomotor processing irrespective of processing demands and the hand used. Potential differences between conditions and their dependency on the hand used were identified based on F-statistics (main effects and interactions) and subsequent one sample t-tests between pairs of conditions (e.g. ST_Rhand_ + ST_Lhand_ > T_Rhand_ + T_Lhand_). The resulting statistical parametric maps were thresholded at *p* < 0.05 corrected for multiple comparisons on the voxel level (family wise error, FWE) and viewed within the respective model-specific inclusive mask. Cluster sizes of activations (k) are provided in the Tables.

#### Activation asymmetries between hemispheres

Condition-specific lateralization was assessed by flipping first-level contrast images of interest (condition against baseline) and comparing flipped with unflipped images voxel-wise in an additional random effects analysis (Hand x Condition (flipped/unflipped) ANOVA). A midline mask was used to exclude voxels whose intensity may have been affected by the flipping procedure [53]. Effector-independent activation asymmetries were identified according to previously described procedures [16, 54]. This excluded the possibility that lateralization effects were driven by different shapes of the HFR in homologous regions.

Hand-independent lateralization was assessed by means of a conjunction analysis over the condition-specific lateralization maps of the right and left hand respectively ([Cond_Rhand_ > _flip_Cond_Rhand_ ∩ Cond_Lhand_ > _flip_Cond_Lhand_], *p* < 0.05 FWE corrected). This analysis revealed those brain areas that were significantly lateralized for both hands (and not only one). It was thus ensured that a lateralized effect was not just a consequence of the contralateral organization of the visual or motor system but represented hemispheric specialization independent of visual hemifields and hands [16, 54]. Within these condition-specific lateralization masks, we tested whether lateralized effects were greater in one condition relative to the others by voxel-wise comparison of hemispheric activation differences between conditions (e.g. [(ST_Rhand_ > _flip_ST_Rhand_) > (T_Rhand_ > _flip_T_Rhand_) ∩ (ST_Lhand_ > _flip_ST_Lhand_) > (T_Lhand_ > _flip_T_Lhand_)], *p* < 0.05 FWE small volume corrected).

Hand-specific lateralization was assessed by comparing flipped and unflipped images of one and the same hand within a condition (e.g. Cond_Rhand_ > _flip_Cond_Rhand_). Effector-independent lateralization was masked out. This analysis revealed whether activation was higher in one hemisphere relative to the other for either the right or the left hand. The threshold for significance was set at *p* < 0.05 FWE corrected for multiple comparisons. Statistical parametric maps were viewed within the respective model-specific mask. Please note that asymmetrical masking of statistical parametric maps could not have generated lateralized effects, since such a procedure does not affect statistical values of a contrast and is performed after the actual statistical test.

Results were visualized using MRIcro [55]. Coordinates are given in MNI space. Anatomic functional inference was based on probabilistic maps of the SPM anatomy toolbox [56] or the human motor area template [57].

## Results

### Behavioral data

**Fig 2:**
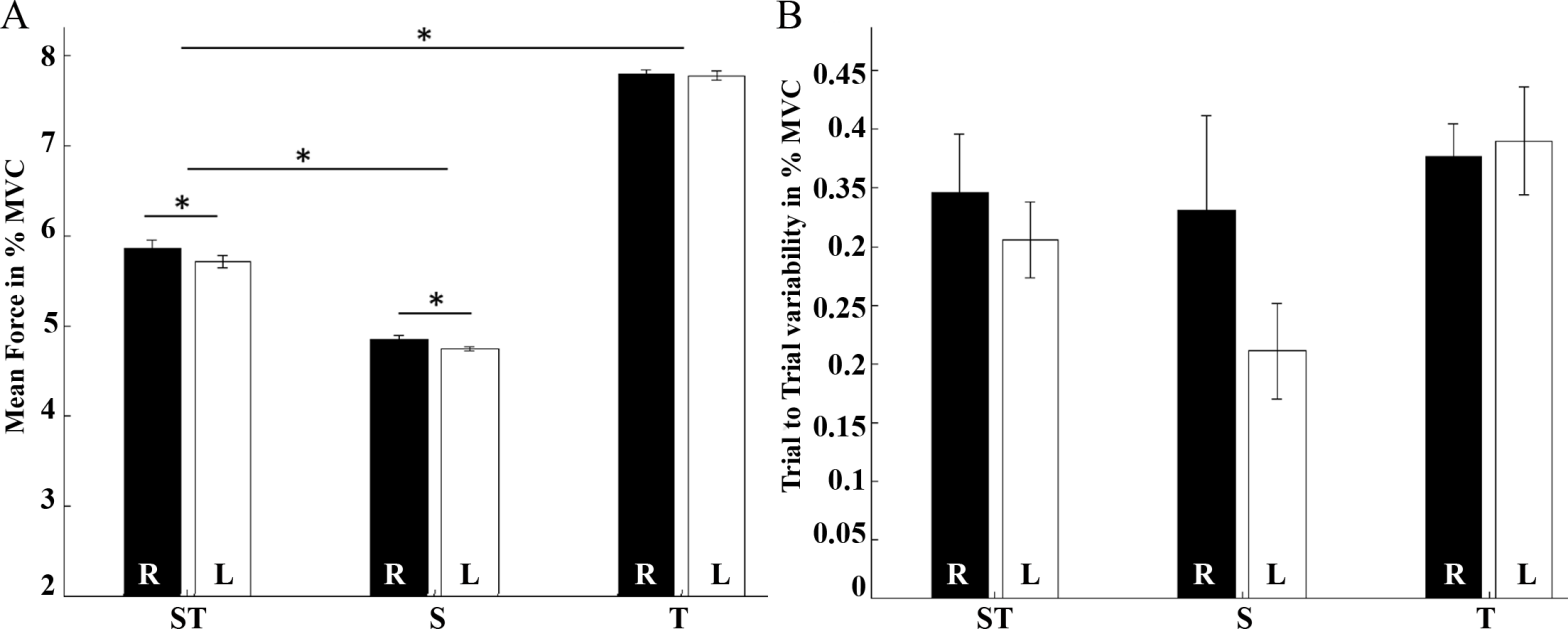
Behavioral data. (A) Mean force and (B) Trial to Trial variability during the high spatial and high temporal (ST-), high spatial and low temporal (S-) and low spatial and high temporal (T-) processing demands condition with the right (black, R) and left (white, L) hand. Error bars indicate standard error in percent maximal voluntary contraction (% MVC). Significant differences (p < 0.001) are marked (*).

Fig 2 and Table 1 display mean force and trial to trial variability for each condition performed with the right and the left hand. To test whether there were any differences between conditions in mean force or trial to trial variability, we performed two 2x3 factorial ANOVAs testing the effect of hand (right/left) and condition (ST, S, T) on mean force and trial to trial variability (Table 2). There was a significant main effect for the factor hand and condition on mean force. The interaction of the two factors was not significant. Though mean force was close to 5% MVC during the two conditions with high spatial processing demands, post-hoc t-tests revealed that mean force was higher during the ST-condition compared to the S-condition [ST > S: *t(18)* = 1.114, *p <* 0.001]. Further, as expected, mean force was higher during the condition with high temporal and low spatial processing demands where the spatial movement of the cursor (and thus force output) was not guided. Mean force was higher for the right compared to the left hand (*t(18)* = 0.86, *p* < 0.001; Fig 2A, Table 1). Trial to trial variability did not differ between conditions or hands. Further, we tested whether there was some kind of motor learning throughout the experiment. Mean force deviation did not significantly change from the first to the last 10 trials of a condition (Table 1).

**Table 1:** Mean force, trial to trial variability and learning depending on condition and hand used.

		Mean Force (% MVC)		Trial to Trial Variability		Mean Force deviation first vs last 10 trials		
Condition	Hand	M	SD	M	SD	M	SD	*t*(df), *p*
ST-Condition	Right	5.861	0.402	0.346	0.218	0.014	0.023	*t*(18) = 2.55, *p* = .02
	Left	5.712	0.296	0.306	0.141	-0.018	0.138	*t*(18) = -0.56, *p* = .578
S-Condition	Right	4.853	0.166	0.331	0.355	0.013	0.032	*t*(18) = 1.75, *p* = .097
	Left	4.742	0.090	0.211	0.177	0.007	0.013	*t*(18) = 2.49, *p* = .023
T-Condition	Right	7.963	0.505	0.377	0.124	0.006	0.028	*t*(18) = 0.92, *p* = .369
	Left	7.785	0.488	0.39	0.177	0.008	0.033	*t*(18) = 1.06, *p* = .304

% MVC = percent maximal voluntary contraction, df = degrees of freedom, M = mean, SD = standard deviation, ST = high spatial, high temporal processing demands, S = high spatial, low temporal processing demands, T = low spatial, high temporal processing demands

**Table 2:** Results of the 2x3 (ST, S, T) factorial ANOVA for mean force and trial to trial variability.

		Mean Force		Trial to Trial Variability	
	(df1, df2)	F	*p*	F	*p*
Hand	(1, 18)	9.476	0.006*	2.8154	0.1106
Condition	(2, 18)	472.84	< 0.001*	1.281	0.273
Hand x Condition	(2, 18)	0.346	0.706	1.121	0.337

* significant

df1 = degrees of freedom numerator, df2 = degrees of freedom denominator

### fMRI data

#### Planning of the cursor movement

**Fig 3:**
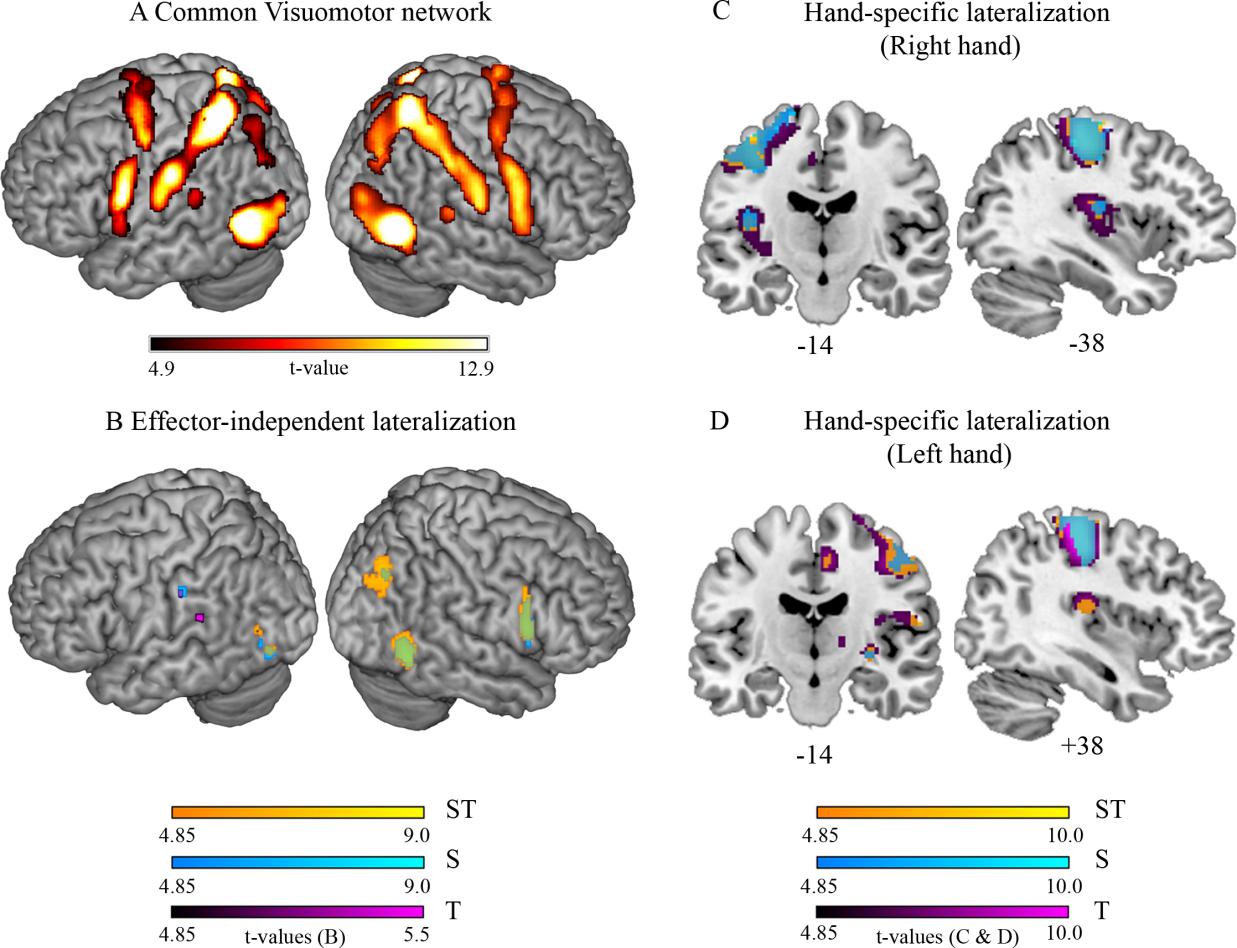
Visuomotor network associated with the planning of virtual avatar movements and its lateralization. (A) Conjunction analysis over all conditions performed with the right and the left hand. (B) Lateralized condition-specific activations that occur irrespective of the effector (CondRHand > flipCondRHand ∩ CondLHand > flipCondLHand). (C & D) Areas that activate more strongly in one hemisphere compared to the other during planning of the virtual avatar movement with either the right (C) or the left (D) hand. Effector-independent activations are masked out. Colors represent the different conditions and their overlap (high spatial, high temporal processing demands (ST) in yellow; high spatial, low temporal processing demands (S) in blue; low spatial, high temporal processing demands (T) in purple; overlay ST ∩ S in green). Significant effects (*p*_FWE_ < 0.05 on the voxel level) are overlaid on a representative brain normalized to MNI space.

The common network associated with visuomotor planning, i.e. the time interval where the reference trajectories were already seen, but movement not yet required, irrespective of the upcoming trajectory and the hand used (ST_Rhand_ ∩ ST_Lhand_ ∩ S_Rhand_ ∩ S_Lhand_ ∩ T_Rhand_ ∩ T_Lhand_) is depicted in Fig 3A and listed in Table 3. Planning of a cursor movement was associated with bilateral activation in PMd, PMv, SMA, CMA, IPL, SPL, the temporo-parietal junction (TPJ) and visual areas (middle occipital and temporal gyri, V4, V5/MT). Further, planning-related activity was observed in the right putamen, caudate nucleus, and thalamus, left cerebellum lobule VI and the cerebellar vermis. Of note, in these latter areas activity was not only observed during visuomotor planning but also during online visuomotor processing (see S1 Table for activity that persists throughout planning and execution). Depending on the hand used, additional activation was observed in contralateral M1, the CMA, globus pallidus, thalamus and the ipsilateral cerebellum lobule V (Table 4).

**Table 3:** Results of the conjunction analysis over all conditions in the planning model.

Anatomical region	BA	k	L/R	x	y	z	*t*
Middle occipital gyrus (LOC)	18	1293	L	-28	-88	6	10.6
Middle occipital gyrus (V5/MT)	37		L	-42	-68	2	19.4
Middle occipital gyrus (LOC)	18/19	1569	R	32	-84	14	9.66
Middle occipital gyrus (LOC)	18		R	38	-84	4	9.44
Middle temporal gyrus (V5/MT)	37		R	46	-66	0	22.6
Superior parietal lobule (7PC)	7	2941	L	-26	-54	60	18.3
Inferior parietal lobule (IPL)	40		L	-54	-26	38	14
Postcentral gyrus	2		L	-32	-38	48	12.3
Superior parietal lobule (7A)	7	2991	R	20	-56	64	15.8
Superior parietal lobule (5)	5		R	34	-46	58	11.9
Supramarginal gyrus	40		R	40	-36	44	11.8
Superior temporal gyrus (TPJ)	22/40	128	L	-48	-38	22	8.87
Inferior parietal lobule (IPL/TPJ)	40	50	R	64	-32	20	8.57
Precentral gyrus (SMA)	6	4149	L	0	-4	56	14
Precentral gyrus (PMd)	6		L	-36	-10	52	11.4
Rolandic operculum (PMv)	6/44		R	56	6	30	11.5
Precentral gyrus (PMv)	6/44	1004	L	-58	2	28	14.2
Middle cingulate cortex	31	120	L	-12	-24	38	8.09
Middle cingulate cortex	31	31	R	14	-22	38	5.83

Reported local maxima are significant with p_FWE_ < 0.05 at the voxel level. Only the three highest local maxima per cluster are reported.

BA = Brodmann’s area, k = cluster size, L/R = left hemisphere/right hemisphere, 7PC = superior parietal area 7 postero-caudal, 7A = superior parietal area 7 anterior, LOC = lateral occipital cortex, PMd = premotor cortex dorsal, PMv = premotor cortex ventral, SMA = supplementary motor area, TPJ = temporo-parietal junction, V5/MT = visual area V5/middle temporal area

The planning-related visuomotor network was not lateralized except for a small cluster in the left middle occipital gyrus (MOG, MNI: -38–68 0, *t* = 5.42).

**Table 4:** Hand-dependent BOLD signal differences averaged over the three conditions in the planning model.

Anatomical region	BA	k	L/R	x	y	z	*t*	k	L/R	x	y	z	*t*
			Left hand > right hand						Right hand > left hand				
Lingual gyrus	18	678	R	18	-76	-12	10.9	769	L	-20	-80	-14	11.3
Middle occipital gyrus (LOC)	18/19	77	R	30	-78	18	6.32	325	L	-30	-84	18	8.7
Superior parietal lobule (7A)	7		-	-	-	-	-	149	L	-16	-74	52	6.24
Precentral gyrus (M1)	4	1909	R	38	-22	52	27	2048	L	-36	-26	56	23.4
Rolandic operculum (OP3)	43	149	R	42	-18	18	10.5	167	L	-38	-20	16	8.42
Middle cingulate cortex (CMA)	24	206	R	8	-8	50	8.69	89	L	-6	-10	48	8.22
Pallidum			-	-	-	-	-	8	L	-24	-8	-4	6.08
Putamen		11	R	30	-4	-2	5.93		-	-	-	-	-
Thalamus		68	R	18	-20	4	8.95	166	L	-16	-22	2	9.47

Reported local maxima are significant with p_FWE_ < 0.05 at the voxel level. Only the three highest local maxima per cluster are reported.

BA = Brodmann’s area, k = cluster size, L/R = left hemisphere/right hemisphere, 7A = superior parietal area 7 anterior, CMA = cingulate motor area, LOC = lateral occipital cortex, M1 = primary motor cortex, OP3 = ventral anterior parietal operculum

**Effector-independent, right-lateralized effects for the ST-, S- and T-condition**

The condition-dependent lateralization analysis revealed effector-independent signal asymmetries (Table 5, Fig 3B). When the reference trajectory was associated with high spatial processing demands (yellow (ST-condition) and blue (S-condition) clusters in Fig 3B, overlap in green), there was right-lateralized activity in the inferior occipital and temporal gyri (V5/MT), the middle occipital gyrus (MOG) at the border to the inferior parietal lobe and the PMv. Activity in the CMA (MNI: 8 16 24) was marginally right-lateralized (not illustrated) when high spatial processing demands were accompanied by high temporal processing demands (*t* = 4.79, *p*_FWE_ = 0.062). The right-lateralized effects were independent of the hand to be used.

**Table 5:** Brain regions exhibiting effector-independent lateralized BOLD responses during visuomotor planning.

Anatomical region	BA	k	L/R	x	y	z	*t*	k	L/R	x	y	z	*t*	k	L/R	x	y	z	*t*
			ST-condition						S-condition						T-condition				
Superior occipital gyrus	18		-	-	-	-	-	15	L	-22	-70	28	6.09	13	L	-20	-78	32	5.35
Middle occipital gyrus	19	29	L	-38	-68	2	5.81	11	L	-38	-68	0	5.52	26	L	-38	-68	2	6.11
Middle occipital gyrus	19	109	R	34	-72	38	6.62	7	R	34	-70	38	5.08	12	R	34	-68	26	5.55
Middle occipital gyrus	19		R	32	-82	36	5.74		-	-	-	-	-		-	-	-	-	-
Inferior occipital gyrus (LOC)	19	7	L	-46	-78	-6	5.24	13	L	-48	-78	-6	5.51		-	-	-	-	-
Middle temporal gyrus	37	271	R	46	-60	0	7.96	173	-	-	-	-	-		-	-	-	-	-
Inferior temporal gyrus	37		R	54	-58	-6	8.77		R	52	-60	-8	7.44		-	-	-	-	-
Supramarginal gyrus (SMG)	40		-	-	-	-	-	9	L	-52	-26	34	5.68	2	L	-54	-26	36	5.01
Superior temporal gyrus (TPJ)	22/40		-	-	-	-	-	5	L	-46	-36	20	5.18	32	L	-48	-38	20	6.46
Inferior frontal gyrus (PMv)	44	176	R	58	12	14	7.1	132	R	56	12	14	6.86		-	-	-	-	-
Inferior frontal gyrus (PMv)	44		R	54	12	26	6.7		R	56	12	26	5.57		-	-	-	-	-

Reported local maxima are significant with *p*_FWE_ < 0.05 at the voxel level. Only the three highest local maxima per cluster are reported.

BA = Brodmann’s area, k = cluster size, L/R = left hemisphere/right hemisphere, LOC = lateral occipital cortex, PMv = premotor cortex ventral, TPJ = temporo-parietal junction

**Effector-independent, left-lateralized effects**

During planning, effector-independent left-lateralized BOLD responses were observed in the inferior and middle occipital gyrus (V5/MT) for all three conditions (yellow (ST-condition), blue (S-condition) and purple (T-condition) clusters in Fig 3B, overlay S- and ST-condition in green). Further, BOLD responses in the supramarginal gyrus (SMG) were left lateralized for the S- and T-condition. Activity in the TPJ was only significantly left-lateralized when spatial processing demands were low and temporal demands high (purple (T-condition) cluster in Fig 3B).

**Lateralization differences between conditions**

Next, we analyzed the extent to which interhemispheric activation asymmetries differed between the three conditions. We tested whether right-lateralized effects were greater for high than low spatial processing demands [(ST/S_Rhand_ > _flip_ST/S_Rhand_) > (T_Rhand_ > _flip_T_Rhand_) ∩ (ST/S_Lhand_ > _flip_ST/S_Lhand_) > (T_Lhand_ > _flip_T_Lhand_)] and whether left-lateralized effects were greater when spatial processing demands decreased while temporal processing demands increased [(T_Rhand_ > _flip_T_Rhand_) > (ST/S_Rhand_ > _flip_ST/S_Rhand_) ∩ (T_Lhand_ > _flip_T_Lhand_) > (ST/S_Lhand_ > _flip_ST/S_Lhand_)]. Right lateralization was greater during planning of the ST-condition relative to the T-condition in the inferior temporal gyrus [MNI: 52–56–8 *t* = 4.98; MNI: 54–60–2 *t* = 4.72; MNI: 44–54–2 *t* = 4.75], MOG [MNI: 36–72 34 *t* = 4.03; MNI: 40–74 28 *t* = 4.43], and PMv [MNI: 54 10 20 *t* = 5.32]. With exception of the MOG, the same was observed for the S-condition relative to the T-condition [MNI_MTG_: 44–54–2 *t*_MTG_ = 3.83; MNI_ITG_: 54–58–6 *t*_ITG_ = 3.71; MNI_PMv_: 52 10 16 *t*_PMv_ = 4.58]. There were no significant left-lateralized condition differences in the CMA, the SMG, and the TPJ.

**Hand-specific lateralized effects**

To test whether activation was higher in one hemisphere relative to the other for virtual avatar control by either the right or the left hand, we assessed hand-specific BOLD signal asymmetries (S2 Table, Fig 3C and 3D). Planning of almost all conditions was associated with activation lateralized to contralateral visual areas, M1, S1, CMA, rolandic operculum, posterior insula, thalamus, the putamen, and ipsilateral cerebellum lobule V and VI. Lateralized effects in the cerebellum were observed during visuomotor planning and online visuomotor processing.

#### Online visuomotor processing

**Fig 4:**
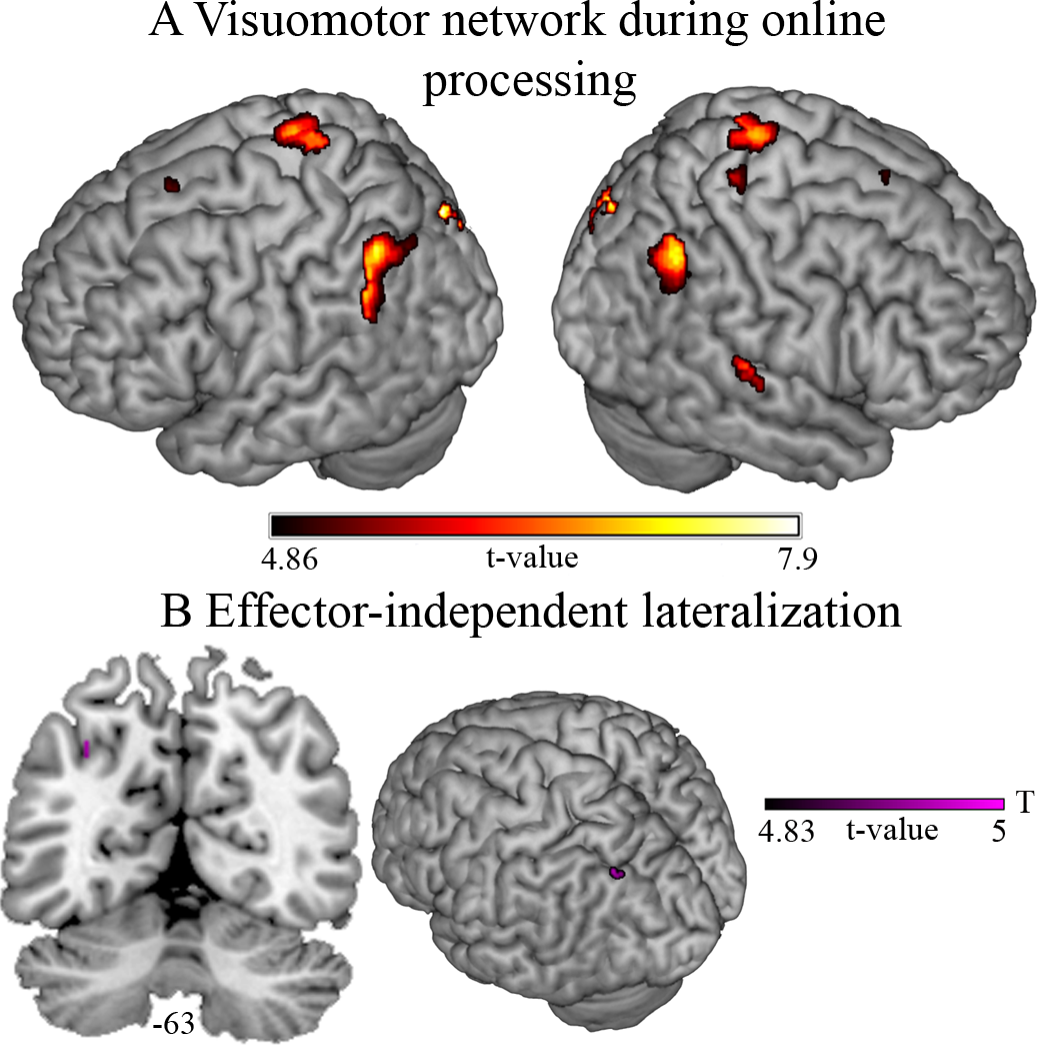
Online visuomotor processing. (A) Visuomotor network associated with the ongoing virtual avatar movement as revealed by a conjunction analysis over all conditions performed with the right and left hand. (B) Result of the conjunction analysis revealing the left intraparietal sulcus as the only region that activated more strongly in one relative to the other hemisphere, irrespective of the effector (Cond_RHand_ > flipCond_RHand_ ∩ Cond_LHand_ > flipCond_LHand_) in the low spatial, high temporal (T-) processing demands condition (purple). Significant voxels (p_FWE_ < 0.05 at the voxel level) are overlaid on a representative brain normalized to MNI space.

The common network during the ongoing cursor movement (ST_Rhand_ ∩ ST_Lhand_ ∩ S_Rhand_ ∩ S_Lhand_ ∩ T_Rhand_ ∩ T_Lhand_) was associated with activation in bilateral IPL, IPS, S1, the middle frontal gyrus (MFG), and the right superior temporal gyrus (STG) at the border of the SMG (Fig 4A, Table 6). Further, activity was observed in the right putamen, caudate nucleus, and thalamus, left cerebellum lobule VI, the cerebellar vermis and ipsilateral cerebellum lobule V, where activity was already present during visuomotor planning (see S1 Table for details). The common online visuomotor network was not lateralized. The condition-specific lateralization analysis revealed effector-independent left lateralization in the IPS during the T-condition (MNI: -32–66 40, *t* = 5.03; purple cluster in Fig 4B). The analysis of lateralization differences between conditions revealed lateralized effects in the IPS were not statistically different between conditions. Hand-specific lateralization was only observed in the ipsilateral cerebellum lobule V and VI where lateralization was also observed during visuomotor planning (see S2 Table). Of note, contralateral M1 activity was not lateralized in a hand-specific manner, since activity in this region was better predicted by the planning model and thus masked out in the online visuomotor processing model.

**Table 6:** Results of the conjunction analysis over all conditions during online visuomotor processing.

Anatomical region	BA	k	L/R	x	y	z	*t*
Cuneus (V3d)	18	428	L	-4	-84	32	9.85
Cuneus (V3d)	19		R	8	-82	34	10
Superior parietal lobule (5Ci)	5	9	R	2	-32	44	5.29
Inferior parietal lobule (IPL)	40	271	L	-46	-52	50	7.34
Inferior parietal lobule (IPL)	40		L	-58	-46	38	6.94
Intraparietal sulcus (IPS)	40		L	-38	-56	42	6.19
Inferior parietal lobule (IPL)	40	204	R	48	-52	44	7.24
Superior temporal gyrus	22	27	R	58	-24	8	6.32
Superior temporal gyrus	22		R	62	-18	2	5.57
Postcentral gyrus (S1)	3	23	R	20	-36	64	5.89
Paracentral lobule (M1/S1)	4/1	215	R	2	-30	-74	6.86
Paracentral lobule (M1/S1)	4/1		R	-6	-38	74	6.44
Superior frontal gyrus (pre-SMA)	8	15	L	-2	18	58	5.23

Reported local maxima are significant with *p*_FWE_ < 0.05 at the voxel level. Only the three highest local maxima per cluster are reported.

BA = Brodmann’s area, k = cluster size, L/R = Left hemisphere/right hemisphere, 5Ci = superior parietal area 5 around cingulate sulcus, M1 = primary motor cortex, pre-SMA = presupplementary motor area, S1 = primary somatosensory cortex, V3d = visual area 3 dorsal

#### Overall condition differences

For completeness, condition differences independent of their lateralization are provided in S1 Fig and S3 Table. Areas displaying greater right-lateralization when spatial processing demands are high (posterior temporal gyrus, MOG, PMV) also display higher activation in the right hemisphere during the ST-condition relative to the T-condition (yellow ST > T and green (ST > T and S > T cluster in S1 Fig). During online visuomotor processing, the only condition difference in non-visual areas concerned the left putamen (not illustrated). Here, activity was higher when spatial processing demands were low and temporal demands high [T > ST: MNI: -24 4–10, *t* = 7.44].

## Discussion

The present study examined asymmetries in hemispheric activation during visuomotor control of a virtual avatar. In particular, it was examined how lateralization in the underlying neural network changed depending on visuospatial and visuotemporal processing demands that result from condition differences in the virtual avatar’s reference trajectory. We additionally separated effects of visuomotor planning from online visuomotor processing by investigating trial phases separately. Planning of a virtual avatar movement activated a bilateral visuomotor network, expectedly. In this network, increasing spatial processing requirements were associated with greater involvement of right lateralized cortical areas involved in visuomotor processing including the posterior ITL (V4, V5/MT), MOG, and PMv. Increasing temporal processing demands, on the other hand, were associated with left lateralized activation in the TPJ during planning. During online visuomotor processing, when participants operated the virtual avatar, right lateralization was not observed. Instead, left lateralized activity was observed in the IPS. The present findings imply that input-related spatiotemporal processing requirements affect hemispheric asymmetries during visuomotor planning. Our data suggest that the right hemisphere is preferentially involved in perceptual analyses that support the planning of spatial movement features, whereas the left hemisphere is preferentially concerned with movement timing indicated by visual cues. Further, during an actual movement, the left relative to the right hemisphere may monitor and adjust avatar trajectories with respect to both temporal and fine spatial aspects.

### Interpretational issues

#### Lateralization

The lateralization effects observed here represent relative BOLD signal differences between two homotopes, i.e. a brain region and its homologous region in the other hemisphere. These functional asymmetries do not necessarily imply absolute functional specialization. Rather, lateralization effects can be interpreted as a hemisphere’s processing preference for certain aspects within an overall bilateral network. Consequently, hemispheric asymmetries should be interpreted in the sense that the hemispheres differ with regard to their relative contribution to a certain process and do not represent function in one but not the other hemisphere.

#### Trial phase

Further, when interpreting the results, it is important to keep in mind that the methodological approach employed allows one only to decide whether a brain region was more involved in one trial phase compared to the other. This rendered a separation of planning- and online processing-related effects incomplete. Despite this interpretational restriction, the approach provides important information which could not have been obtained by combining temporally-close and non permutable events in a single regressor.

### The common visuomotor network of virtual avatar control

Planning of the virtual avatar movement, irrespective of visuospatial and visuotemporal processing demands, was associated with widespread bilateral cortical and subcortical activation. The conjoint activation of these areas resembled a typical visuomotor network for visually-guided power or precision grip tasks [33, 39, 58, 59] but also resembled the one observed for reaching and pointing [60, 61]. This suggests that most of the neocortical activations in these studies can be attributed to visuomotor processing and are not solely related to movements of the hand in proprioceptive and visual space. While most visuomotor studies reported the network without distinction of planning- and online processing-related effects, the present findings showed that the observed BOLD response in bilateral visual areas (V4, V5/MT), IPL, SPL, PMv, PMd and contralateral M1, CMA, putamen and thalamus was better predicted by regressors that were time-locked to planning rather than to online processing.

### Lateralized effects during visuomotor planning

#### Hand-specific lateralized effects

As expected from the general contralateral organization of the visual and motor system, activity in several visual areas, M1, S1, CMA, posterior insula and thalamus was lateralized to the hemisphere contralateral (in the case of the cerebellum ipsilateral) to the hand which would produce the instructed virtual avatar movement. An alternative possibility that cannot be discounted, due to confounding of hemifield presentation of instructions with response hand, is that the activity was lateralized to the hemisphere contralateral to the hemifield in which the reference trajectory was presented.

#### Effector-independent lateralized effects

The main finding of the present study was that higher spatial processing demands during evaluation of a visual reference trajectory for motor planning were associated with right-lateralized brain activity. Specifically, the BOLD signal in V4, V5/MT, MOG, and PMv was higher in the right than the left homotope when visual information indicated the expected vertical cursor deflection (high spatial processing demands, ST- and S-condition). When visual information indicated only a spatially unspecified deflection of the cursor from the home position (low spatial processing demands: T-condition), right lateralization was drastically reduced. Right lateralization was independent of the hand used and the visual field in which the stimulus was presented. For this reason, it can be concluded that the observed right lateralization reflected a hemispheric preference for visuomotor processing and was not a mere by-product of the general contralateral organization of the visual or motor system.

The right hemisphere’s role for spatial processing during visuomotor planning was predicted given its role in perception. A relationship between the right hemisphere and spatial processing has previously only been described during visuomotor tasks with high spatial and high temporal processing demands [16]. Consequently, the previously observed right-lateralized effects during visuomotor processing could also have been related to temporal processing. Based on the present findings, we conclude that the right hemisphere preferentially processes spatial aspects of sensory information during visuomotor planning. Concordant with a planning-dependent right hemisphere processing preference for spatial information, damage to the right but not left hemisphere prolongs reaction times (indicative of impaired motor planning) for visually guided reaches, whereas the actual movement is unimpaired [62].

Complementary to the right hemisphere’s processing preference for spatial information during visuomotor planning, our data suggest that the left hemisphere is relatively more involved in temporal processing of sensory information. In particular, we observed hand-independent, left-lateralized planning-related activity in area V5/MT, the TPJ and the SMG especially when spatial processing demands were low and temporal processing demands high (T-condition). Area V5/MT, the TPJ, and SMG have been associated with temporal estimation of visual events, i.e., indicating the moment when a visual event occurs [63, 64] or for how long a visual stimulus is presented [65, 66]. Therefore, we assume that the observed activity in these regions was related with the extraction of temporal information from visual input to predict the appropriate timing of the cursor deflection and thus movement onset. The lack of significant differences between the T- and S-condition confirms that even in the condition with high spatial processing demands movement on- and offset had to be planned.

In summary, our results support the notion that functional asymmetries described in perception, specifically a right hemisphere processing preference for spatial information and left hemisphere processing preference for temporal information [18–24, 31], also apply to visuomotor planning. Research in split-brain patients further suggests that perceptual functional asymmetries may be stronger in a visuomotor context compared to a purely perceptual task [28]. Yet, fMRI studies in perception [21, 22, 65, 66] as well as the current study report lateralized effects within a bilateral task network. Because we did not include another experimental factor in the study design that could disambiguate perceptual processing from output related processing, our results do not allow us to decide whether there are differences in the degree of functional lateralization between perceptual and visuomotor tasks.

### Lateralized effects during online visuomotor processing

During the actual virtual avatar movement, condition- and hand-independent right-lateralization was not observed. Instead, condition- and hand-independent left lateralization was observed in the IPS. We thus assume that this asymmetry reflects a left hemisphere preference for processes serving the online control of the cursor, i.e., online visuomotor processing.

Concordant with this idea, the IPS has been linked with action monitoring and the initiation of corrective responses during ongoing movements. For example, disruptive transcranial magnetic stimulation (TMS) pulses applied to the left IPS just after movement onset prevent smooth hand pathway corrections in response to target displacements [67] or prevent adjustments of reaching movements to external force fields [68]. It has been demonstrated that TMS to the IPS affects hand pathway adjustments only around the time of adjustment initiation and not afterwards [69]. This implies that IPS is more associated with online visuomotor processing in terms of deviation monitoring rather than with error correction implementation. From a behavioral point of view, a left hemispheric online processing preference would be consistent with the observation that damage to the left but not right hemisphere impairs adjustments of ongoing visuomotor movements in terms of, for example, reach [62] or hand aperture scaling precision [70].

The lack of condition differences in left IPS during the ongoing cursor movement suggests that processing of spatial aspects is also left-lateralized during online visuomotor processing. This could be a consequence of the fact that local spatial processing of visual information relies on a higher spatial resolution. It has been suggested that the left relative to the right hemisphere filters sensory information in a relatively higher frequency range allowing for an efficient analysis of local spatial features [25]. This may support the deviance detection between the current and intended virtual avatar position that occurs in the local range of spatial frequencies. Alternatively, the left-lateralized activation of the IPS during online visuomotor control could also be interpreted as a reflection of a general left hemisphere dominance for motor-output related processing [8, 9, 11]. Yet, this would have likely resulted in a lateralization pattern that includes premotor cortices, which was not observed. Further research could distinguish a general left hemisphere online visuomotor processing preference from a sensory input-dependent functional specialization.

Functional studies assessing hemispheric asymmetries in the IPS during reach-to-grasp actions [29, 71] have reported effector-dependent lateralization. During reaching and grasping, the hand moves in (visual but also propioceptive) space in relation to a target. This results in effector-dependency. In contrast, our results indicate that there are additional hemispheric asymmetries in the IPS that are hand-independent but rather related to online visuomotor processing *per se*.

### General discussion

Our results hint at two important contextual factors that affect the relative contribution of the cerebral hemispheres during visuomotor processing: the dissociation between planning and online control on the one hand and the degree of spatial vs. temporal processing demands on the other hand. As indicated by the model comparison approach, right lateralization was especially apparent during planning and became much less prominent when visual information was processed to adjust cursor movement inflight. This supports the notion that hemispheric asymmetries during visuomotor processing are not well described by a general right hemisphere processing preference for sensory information. According to the DFF theory [25] the right hemisphere acts as a low pass filter for visuospatial information which leads to a rather global representation of visual information, which allows for an efficient analysis of visuospatial features. Such an analysis would mainly support visuomotor planning and not necessarily online visuomotor processing, during which the CNS would need to extract more local features (see above). Thus, processing visual information more globally in the right hemisphere and more locally in the left hemisphere may cause right lateralization of spatial processing during visuomotor planning and left lateralization of spatial processing during online visuomotor processing.

During visuomotor processing, empirical evidence regarding a relationship between the right hemisphere and visuospatial processing has been inconsistent. In particular studies investigating reach-to-grasp actions by means of fMRI have often failed to detect right lateralized effects [29, 30, 72]. Our results imply a role of planning-related processes for right lateralization. Thus, tasks with low demands on planning may fail to reveal right lateralization. Alternatively, the right lateralization observed in this experiment may constitute a consequence of the artificial mapping of visuospatial information to isometric force / proprioception. Such a novel association is not required during reach-to-grasp actions. Right lateralization as observed here could thus relate to increased processing demands due to an incongruent mapping between proprioception and cursor movement in visual space. Yet, the right lateralization was observed during planning only, during which no movement took place and during which proprioceptive feedback did not yet play a role. Consequently, any effect that would have been generated by the discrepancy between the virtual avatar movement and the hand movement should have occurred during online visuomotor control.

One could be tempted to characterize activity in the aforementioned lateralized areas as related to purely sensory, motor or sensorimotor processes. Yet, in the light of recent research pointing towards a direct link between perception and action, a clear distinction between purely sensory, motor, or sensorimotor processes seems increasingly unlikely. For example, studies have shown that seemingly ‘purely’ perceptual tasks concern the motor system [73, 74] and evidence regarding an influence of perception on action is manifold [5, 75]. For this reason, it was not the scope of the present study to isolate perceptual and motor processes. Instead, our findings are valuable since they demonstrate how functional asymmetries are changed by the way visual information is processed during a rather common visuomotor task, the control of a cursor.

### Limitations

Because fixation was only instructed but not objectively controlled, effects of eye movement cannot be entirely ruled out. Given that the effects of interest were observed independent of the visual hemifield in which the reference trajectory was presented, we believe eye movement effects were marginal.

Behavioral data showed that mean force was not identical between the three conditions. The maximum difference in % MVC of 2–3% between conditions may have influenced the BOLD response in contralateral primary motor/somatosensory cortices and the ipsilateral cerebellum that linearly reflect the level of produced force [76–78]. Indeed, activity in contralateral M1 and the ipsilateral cerebellum was stronger for the T-condition (where mean force was highest) compared to the other two conditions, which suggests that force could have affected activity in these areas. Importantly, effector-independent condition differences did not concern brain areas associated with a linear relationship between force and the BOLD signal. Thus, our main findings are not influenced by differences in % MVC between conditions.

The robustness of the study could have been improved by increasing the number of trials for each condition. Yet, this could have affected performance measures which was not the case in our study.

The present study provided only description of activation differences between hemispheres and did not reveal the underlying mechanisms of right/left hemisphere processing preferences. Yet, the differences here observed can be taken as a starting point to further characterize the mechanisms underlying functional asymmetries. This should not only be done in terms of an even more exact specification of task-related characteristics influencing relative activation asymmetries between the hemispheres, but also with respect to context dependent intra- and interhemispheric functional connections proposed to govern more general hemispheric specializations [79, 80].

### Conclusions

The present study investigated the contribution of spatial and temporal processing demands during visuomotor planning and online visuomotor processing to hemispheric activation asymmetries when controlling virtual avatar movements. Contrary to the view of a general right hemisphere preference for input-related sensory processing and a left hemisphere preference for output-related motor functions, the findings imply a more fine grained distinction in which both hemispheres contribute to the processing of visual information. Specifically, our results suggest that within a bilateral visuomotor system, the right hemisphere contributes especially to spatial processing of global sensory information to plan spatial movement features of the virtual avatar whereas the left hemisphere contributes primarily to the temporal processing of sensory information to plan movement timing. Functional lateralization differs when the movement is actually executed. During online control of the virtual avatar, the left hemisphere preferentially contributes to more local processing of sensory information allowing fine spatial and temporal adjustments.

Our results suggest dynamic contributions of both hemispheres and provide thus a more complex picture compared to lesion studies. Future research on hemispheric specialization should especially focus on the rich dynamics of intra- and interhemispheric interactions, preferably using methods with higher temporal resolution.

## Supporting information

S1 Video Visual example of the virtual avatar control task. (MP4)

S1 Fig Planning-related BOLD signal differences between conditions as revealed by pairwise comparisons between the conditions. Upper row: Effector-independent differences between conditions during planning. Lower rows: Hand-specific differences between conditions during planning. Differences that were hand-nonspecific are masked out. Colors represent the different pairwise comparisons contrasts and their overlap (high spatial, high temporal processing demands (ST) in yellow; high spatial, low temporal processing demands (S) in blue; low spatial high temporal processing demands (T) in purple; overlay ST ∩ S in green). SPMs are overlaid on a representative brain normalized to MNI space (p_FWE_ < 0.05 on the voxel level).
(TIF)

S1 Table Results of the conjunction analysis over all conditions and hand-dependent BOLD signal differences averaged over the three conditions in the combined model. (DOCX)

S2 Table Brain regions exhibiting hand-specific lateralized BOLD responses. (DOCX)

S3 Table Brain regions exhibiting different BOLD responses depending on condition. (DOCX)

## References

[1] Kawato M. Internal models for motor control and trajectory planning. Curr Opin Neurobiol. 1999;9(6):718–27. 10607637 10.1016/s0959-4388(99)00028-8

[2] Desmurget M, Grafton S. Forward modeling allows feedback control for fast reaching movements. Trends Cogn Sci. 2000;4(11):423–31. 11058820 10.1016/s1364-6613(00)01537-0

[3] Prablanc C, Echallier JE, Jeannerod M, Komilis E. Optimal response of eye and hand motor systems in pointing at a visual target. II. Static and dynamic visual cues in the control of hand movement. Biol Cybern. 1979;35(3):183–7. 518938 10.1007/BF00337063

[4] Riemann BL, Lephart SM. The Sensorimotor System, Part II: The Role of Proprioception in Motor Control and Functional Joint Stability. J Athl Train. 2002;37(1):80–4. 16558671 PMC164312

[5] Friston K. What is optimal about motor control? Neuron. 2011;72(3):488–98. doi: 10.1016/j.neuron.2011.10.018 22078508 10.1016/j.neuron.2011.10.018

[6] Rotella MF, Nisky I, Koehler M, Rinderknecht MD, Bastian AJ, Okamura AM. Learning and generalization in an isometric visuomotor task. J Neurophysiol. 2015;113(6):1873–84. doi: 10.1152/jn.00255.2014 25520430 10.1152/jn.00255.2014

[7] Vaillancourt DE, Thulborn KR, Corcos DM. Neural basis for the processes that underlie visually guided and internally guided force control in humans. J Neurophysiol. 2003;90(5):3330–40. doi: 10.1152/jn.00394.2003 12840082 10.1152/jn.00394.2003

[8] Haaland KY, Elsinger CL, Mayer AR, Durgerian S, Rao SM. Motor sequence complexity and performing hand produce differential patterns of hemispheric lateralization. J Cogn Neurosci. 2004;16(4):621–36. doi: 10.1162/089892904323057344 15165352 10.1162/089892904323057344

[9] Kimura D, Archibald Y. Motor functions of the left hemisphere. Brain. 1974;97:337–50 4434181 10.1093/brain/97.1.337

[10] Chen R, Gerloff C, Hallett M, Cohen LG. Involvement of the ipsilateral motor cortex in finger movements of different complexities. Ann Neurol. 1997;41(2):247–54. doi: 10.1002/ana.410410216 9029074 10.1002/ana.410410216

[11] Hammond GR, Fox AM. Electrophysiological evidence for lateralization of preparatory motor processes. Neuroreport. 2005;16(6):559–62. 15812307 10.1097/00001756-200504250-00008

[12] Frak V, Bourbonnais D, Croteau I, Cohen H. Interlimb transfer of grasp orientation is asymmetrical. ScientificWorldJournal. 2006;6:1805–9. doi: 10.1100/tsw.2006.291 17195876 10.1100/tsw.2006.291 PMC5917298

[13] Frey SH. Tool use, communicative gesture and cerebral asymmetries in the modern human brain. Philos Trans R Soc Lond B Biol Sci. 2008;363(1499):1951–7. Epub 2008/02/23. doi: 10.1098/rstb.2008.0008 18292060 10.1098/rstb.2008.0008 PMC2606701

[14] Corballis MC. The genetics and evolution of handedness. Psychol Rev. 1997;104(4):714–27. 9337630 10.1037/0033-295x.104.4.714

[15] Pohl PS, Luchies CW, Stoker-Yates J, Duncan PW. Upper extremity control in adults post stroke with mild residual impairment. Neurorehabil Neural Repair. 2000;14(1):33–41. doi: 10.1177/154596830001400104 11228947 10.1177/154596830001400104

[16] Callaert DV, Vercauteren K, Peeters R, Tam F, Graham S, Swinnen SP, et al Hemispheric asymmetries of motor versus nonmotor processes during (visuo)motor control. Hum Brain Mapp. 2011;32(8):1311–29. doi: 10.1002/hbm.21110 20681013 10.1002/hbm.21110 PMC6870081

[17] Serrien DJ, Ivry RB, Swinnen SP. Dynamics of hemispheric specialization and integration in the context of motor control. Nat Rev Neurosci. 2006;7(2):160–7. doi: 10.1038/nrn1849 16429125 10.1038/nrn1849

[18] Corballis MC. Mental rotation and the right hemisphere. Brain Lang. 1997;57(1):100–21. doi: 10.1006/brln.1997.1835 9126409 10.1006/brln.1997.1835

[19] Corballis PM. Visuospatial processing and the right-hemisphere interpreter. Brain Cogn. 2003;53(2):171–6. 14607141 10.1016/s0278-2626(03)00103-9

[20] Coull JT, Nobre AC. Where and When to Pay Attention: The Neural Systems for Directing Attention to Spatial Locations and to Time Intervals as Revealed by Both PET and fMRI. J Neurosci. 1998;18(18):7426–35 9736662 10.1523/JNEUROSCI.18-18-07426.1998 PMC6793260

[21] Milivojevic B, Hamm JP, Corballis MC. Functional neuroanatomy of mental rotation. J Cogn Neurosci. 2009;21(5):945–59. doi: 10.1162/jocn.2009.21085 18702586 10.1162/jocn.2009.21085

[22] Williams SC, Brammer MJ, Bullmore ET, Suckling J. Hemispheric preference in visuospatial processing: a complementary approach with fMRI and lesion studies. Hum Brain Mapp. 2000;10(2):80–6. 10864232 10.1002/(SICI)1097-0193(200006)10:2<80::AID-HBM40>3.0.CO;2-2 PMC6871993

[23] Corballis MC. Hemispheric interactions in temporal judgements about spatially separated stimuli. Neuropsychology. 1996;10:42–50.

[24] Nicholls ME, Gora J, Stough CK. Hemispheric asymmetries for visual and auditory temporal processing: an evoked potential study. Int J Psychophysiol. 2002;44(1):37–55. 11852156 10.1016/s0167-8760(01)00190-8

[25] Ivry RB, Robertson LC. The Two Sides of Perception: MIT Press; 1997.

[26] Deruelle C, Fagot J. Hemispheric lateralisation and global precedence effects in the processing of visual stimuli by humans and baboons (Papio papio). Laterality. 1997;2(3–4):233–46. doi: 10.1080/713754268 15513066 10.1080/713754268

[27] Hopkins WD. Hemispheric specialization for local and global processing of hierarchical visual stimuli in chimpanzees (Pan troglodytes). Neuropsychologia. 1997;35(3):343–8. 9051682 10.1016/s0028-3932(96)00089-9

[28] Gazzaniga MS, Bogen JE, Sperry RW. Observations on visual perception after disconnexion of the cerebral hemispheres in man. Brain. 1965;88(2):221–36. 5828904 10.1093/brain/88.2.221

[29] Begliomini C, Sartori L, Miotto D, Stramare R, Motta R, Castiello U. Exploring manual asymmetries during grasping: a dynamic causal modeling approach. Front Psychol. 2015;6:167 doi: 10.3389/fpsyg.2015.00167 25759677 10.3389/fpsyg.2015.00167 PMC4338815

[30] Culham JC, Cavina-Pratesi C, Singhal A. The role of parietal cortex in visuomotor control: what have we learned from neuroimaging? Neuropsychologia. 2006;44(13):2668–84. doi: 10.1016/j.neuropsychologia.2005.11.003 16337974 10.1016/j.neuropsychologia.2005.11.003

[31] Goodale MA. Brain Asymmetries in the Control of Reaching In: Goodale MA, editor. Vision and Action: The Control of Grasping. New Jersey: Ablex Publishing; 1990 p. 14–32.

[32] King M, Rauch HG, Stein DJ, Brooks SJ. The handyman's brain: A neuroimaging meta-analysis describing the similarities and differences between grip type and pattern in humans. Neuroimage. 2014;102 P2:923–37. doi: 10.1016/j.neuroimage.2014.05.064 24927986 10.1016/j.neuroimage.2014.05.064

[33] Ward NS, Frackowiak RS. Age-related changes in the neural correlates of motor performance. Brain. 2003;126(Pt 4):873–88. 12615645 10.1093/brain/awg071 PMC3717766

[34] Begliomini C, Nelini C, Caria A, Grodd W, Castiello U. Cortical activations in humans grasp-related areas depend on hand used and handedness. PLoS One. 2008;3(10):e3388 doi: 10.1371/journal.pone.0003388 18846222 10.1371/journal.pone.0003388 PMC2561002

[35] Martin K, Jacobs S, Frey SH. Handedness-dependent and -independent cerebral asymmetries in the anterior intraparietal sulcus and ventral premotor cortex during grasp planning. Neuroimage. 2011;57(2):502–12. doi: 10.1016/j.neuroimage.2011.04.036 21554968 10.1016/j.neuroimage.2011.04.036 PMC3114104

[36] Gonzalez CL, Whitwell RL, Morrissey B, Ganel T, Goodale MA. Left handedness does not extend to visually guided precision grasping. Exp Brain Res. 2007;182(2):275–9. doi: 10.1007/s00221-007-1090-1 17717653 10.1007/s00221-007-1090-1

[37] Grosskopf A, Kuhtz-Buschbeck JP. Grasping with the left and right hand: a kinematic study. Exp Brain Res. 2006;168(1–2):230–40. doi: 10.1007/s00221-005-0083-1 16078023 10.1007/s00221-005-0083-1

[38] Kuhtz-Buschbeck J, Gilster R, Wolff S, Ulmer S, Siebner H, Jansen O. Brain activity is similar during precision and power gripping with light force: An fMRI study. Neuroimage. 2008;40:1469–81. doi: 10.1016/j.neuroimage.2008.01.037 18316207 10.1016/j.neuroimage.2008.01.037

[39] Neely KA, Coombes SA, Planetta PJ, Vaillancourt DE. Segregated and overlapping neural circuits exist for the production of static and dynamic precision grip force. Hum Brain Mapp. 2013;34(3):698–712. doi: 10.1002/hbm.21467 22109998 10.1002/hbm.21467 PMC3292669

[40] Sterr A, Shen S, Kranczioch C, Szameitat AJ, Hou W, Sorger B. fMRI effects of task demand and feedback accuracy on grip force tracking. Neurosci Lett. 2009;457(2):61–5. doi: 10.1016/j.neulet.2009.04.013 19429163 10.1016/j.neulet.2009.04.013

[41] Vaillancourt DE, Mayka MA, Corcos DM. Intermittent visuomotor processing in the human cerebellum, parietal cortex, and premotor cortex. J Neurophysiol. 2006;95(2):922–31. doi: 10.1152/jn.00718.2005 16267114 10.1152/jn.00718.2005 PMC2366036

[42] Oldfield RC. The assessment and analysis of handedness: the Edinburgh inventory. Neuropsychologia. 1971;9(1):97–113. 5146491 10.1016/0028-3932(71)90067-4

[43] Sulzer JS, Chib VS, Hepp-Reymond MC, Kollias S, Gassert R. BOLD correlations to force in precision grip: an event-related study. Conf Proc IEEE Eng Med Biol Soc. 2011;2011:2342–6. doi: 10.1109/IEMBS.2011.6090655 22254811 10.1109/IEMBS.2011.6090655

[44] Friston KJ, Holmes AP, Worsley KJ, Poline JB, Frith CD, Frackowiak RS. Statistical Parametric Maps in Functional Imaging: A General Linear Approch. Hum Brain Mapp. 1995;2:189–210.

[45] Mumford JA, Poline JB, Poldrack RA. Orthogonalization of regressors in FMRI models. PLoS One. 2015;10(4):e0126255 doi: 10.1371/journal.pone.0126255 25919488 10.1371/journal.pone.0126255 PMC4412813

[46] Poline JB, Kherif F, Pallier C, Penny W. Contrasts and Classical Inference. London: Elsevier; 2006.

[47] Cohen J, Cohen P. Applied Multiple Regression/Correlation Analysis for the Behavioural Sciences. New Jersey: Erlbaum; 1975.

[48] Lancaster BP. Defining and Interpreting Supressor Effects: Advantages and Limitations. Annual Meeting of the Southwest Educational Research Association; San Antonio, TX, USA1999.

[49] Ludlow L, Klein K. Suppressor Variables: The Difference between 'Is' versus 'Acting As'. Journal of Statistics Education 2014;22(2):1–28.

[50] Erdeniz B, Rohe T, Done J, Seidler RD. A simple solution for model comparison in bold imaging: the special case of reward prediction error and reward outcomes. Front Neurosci. 2013;7:116 doi: 10.3389/fnins.2013.00116 23882174 10.3389/fnins.2013.00116 PMC3715737

[51] Penny W, Holmes AJ. Random-Effects Analysis. London: Elsevier; 2003.

[52] Nichols T, Brett M, Andersson J, Wager T, Poline JB. Valid conjunction inference with the minimum statistic. Neuroimage. 2005;25(3):653–60. doi: 10.1016/j.neuroimage.2004.12.005 15808966 10.1016/j.neuroimage.2004.12.005

[53] Wilke M, Lidzba K. LI-tool: a new toolbox to assess lateralization in functional MR-data. J Neurosci Methods. 2007;163(1):128–36. doi: 10.1016/j.jneumeth.2007.01.026 17386945 10.1016/j.jneumeth.2007.01.026

[54] Woolley DG, Wenderoth N, Heuninckx S, Zhang X, Callaert D, Swinnen SP. Visual guidance modulates hemispheric asymmetries during an interlimb coordination task. Neuroimage. 2010;50(4):1566–77. doi: 10.1016/j.neuroimage.2010.01.012 20079443 10.1016/j.neuroimage.2010.01.012

[55] Rorden C, Brett M. Stereotaxic display of brain lesions. Behav Neurol. 2000;12(4):191–200. 11568431 10.1155/2000/421719

[56] Eickhoff SB, Stephan KE, Mohlberg H, Grefkes C, Fink GR, Amunts K, et al A new SPM toolbox for combining probabilistic cytoarchitectonic maps and functional imaging data. Neuroimage. 2005;25(4):1325–35. doi: 10.1016/j.neuroimage.2004.12.034 15850749 10.1016/j.neuroimage.2004.12.034

[57] Mayka MA, Corcos DM, Leurgans SE, Vaillancourt DE. Three-dimensional locations and boundaries of motor and premotor cortices as defined by functional brain imaging: a meta-analysis. Neuroimage. 2006;31(4):1453–74. doi: 10.1016/j.neuroimage.2006.02.004 16571375 10.1016/j.neuroimage.2006.02.004 PMC2034289

[58] Talelli P, Ewas A, Waddingham W, Rothwell JC, Ward NS. Neural correlates of age-related changes in cortical neurophysiology. Neuroimage. 2008;40(4):1772–81. doi: 10.1016/j.neuroimage.2008.01.039 18329904 10.1016/j.neuroimage.2008.01.039 PMC3715371

[59] Ward NS, Swayne OB, Newton JM. Age-dependent changes in the neural correlates of force modulation: an fMRI study. Neurobiol Aging. 2008;29(9):1434–46. doi: 10.1016/j.neurobiolaging.2007.04.017 17566608 10.1016/j.neurobiolaging.2007.04.017 PMC2568861

[60] Ellermann JM, Siegal JD, Strupp JP, Ebner TJ, Ugurbil K. Activation of visuomotor systems during visually guided movements: a functional MRI study. J Magn Reson. 1998;131(2):272–85. doi: 10.1006/jmre.1998.1379 9571103 10.1006/jmre.1998.1379

[61] Filimon F, Nelson JD, Hagler DJ, Sereno MI. Human cortical representations for reaching: mirror neurons for execution, observation, and imagery. Neuroimage. 2007;37(4):1315–28. doi: 10.1016/j.neuroimage.2007.06.008 17689268 10.1016/j.neuroimage.2007.06.008 PMC2045689

[62] Fisk JD, Goodale MA. The effects of unilateral brain damage on visually guided reaching: hemispheric differences in the nature of the deficit. Exp Brain Res. 1988;72:425–35. 3224652 10.1007/BF00250264

[63] Battelli L, Pascual-Leone A, Cavanagh P. The 'when' pathway of the right parietal lobe. Trends Cogn Sci. 2007;11(5):204–10. doi: 10.1016/j.tics.2007.03.001 17379569 10.1016/j.tics.2007.03.001 PMC3613278

[64] Davis B, Christie J, Rorden C. Temporal order judgments activate temporal parietal junction. J Neurosci. 2009;29(10):3182–8. doi: 10.1523/JNEUROSCI.5793-08.2009 19279255 10.1523/JNEUROSCI.5793-08.2009 PMC3862239

[65] Coull JT, Vidal F, Nazarian B, Macar F. Functional anatomy of the attentional modulation of time estimation. Science. 2004;303(5663):1506–8. doi: 10.1126/science.1091573 15001776 10.1126/science.1091573

[66] Morillon B, Kell CA, Giraud AL. Three stages and four neural systems in time estimation. J Neurosci. 2009;29(47):14803–11. doi: 10.1523/JNEUROSCI.3222-09.2009 19940175 10.1523/JNEUROSCI.3222-09.2009 PMC6666014

[67] Desmurget M, Epstein CM, Turner RS, Prablanc C, Alexander GE, Grafton ST. Role of the posterior parietal cortex in updating reaching movements to a visual target. Nat Neurosci. 1999;2(6):563–7. doi: 10.1038/9219 10448222 10.1038/9219

[68] Della-Maggiore V, Malfait N, Ostry DJ, Paus T. Stimulation of the posterior parietal cortex interferes with arm trajectory adjustments during the learning of new dynamics. J Neurosci. 2004;24(44):9971–6. doi: 10.1523/JNEUROSCI.2833-04.2004 15525782 10.1523/JNEUROSCI.2833-04.2004 PMC6730240

[69] Glover S, Miall RC, Rushworth MF. Parietal rTMS disrupts the initiation but not the execution of on-line adjustments to a perturbation of object size. J Cogn Neurosci. 2005;17(1):124–36. doi: 10.1162/0898929052880066 15701244 10.1162/0898929052880066

[70] Tretriluxana J, Gordon J, Fisher BE, Winstein CJ. Hemisphere specific impairments in reach-to-grasp control after stroke: effects of object size. Neurorehabil Neural Repair. 2009;23(7):679–91. doi: 10.1177/1545968309332733 19411406 10.1177/1545968309332733

[71] Rice NJ, Tunik E, Cross ES, Grafton ST. On-line grasp control is mediated by the contralateral hemisphere. Brain Res. 2007;1175:76–84. Epub 2007/09/25. doi: 10.1016/j.brainres.2007.08.009 17888413 10.1016/j.brainres.2007.08.009 PMC2093953

[72] Frey SH, Vinton D, Norlund R, Grafton ST. Cortical topography of human anterior intraparietal cortex active during visually guided grasping. Brain Res Cogn Brain Res. 2005;23(2–3):397–405. doi: 10.1016/j.cogbrainres.2004.11.010 15820646 10.1016/j.cogbrainres.2004.11.010

[73] Schubotz RI, von Cramon DY. Predicting perceptual events activates corresponding motor schemes in lateral premotor cortex: an fMRI study. Neuroimage. 2002;15(4):787–96. doi: 10.1006/nimg.2001.1043 11906220 10.1006/nimg.2001.1043

[74] Schubotz RI, von Cramon DY. A blueprint for target motion: fMRI reveals perceived sequential complexity to modulate premotor cortex. Neuroimage. 2002;16(4):920–35. 12202080 10.1006/nimg.2002.1183

[75] Binder E, Hagelweide K, Wang LE, Kornysheva K, Grefkes C, Fink GR, et al Sensory-guided motor tasks benefit from mental training based on serial prediction. Neuropsychologia. 2014;54:18–27. doi: 10.1016/j.neuropsychologia.2013.11.018 24321273 10.1016/j.neuropsychologia.2013.11.018 PMC5624496

[76] Cramer SC, Weisskoff RM, Schaechter JD, Nelles G, Foley M, Finklestein SP, et al Motor cortex activation is related to force of squeezing. Hum Brain Mapp. 2002;16(4):197–205. doi: 10.1002/hbm.10040 12112762 10.1002/hbm.10040 PMC6871791

[77] Keisker B, Hepp-Reymond MC, Blickenstorfer A, Meyer M, Kollias SS. Differential force scaling of fine-graded power grip force in the sensorimotor network. Hum Brain Mapp. 2009;30(8):2453–65. doi: 10.1002/hbm.20676 19172654 10.1002/hbm.20676 PMC6871245

[78] van Duinen H, Renken R, Maurits NM, Zijdewind I. Relation between muscle and brain activity during isometric contractions of the first dorsal interosseus muscle. Hum Brain Mapp. 2008;29(3):281–99. doi: 10.1002/hbm.20388 17394210 10.1002/hbm.20388 PMC6870705

[79] Stephan KE, Fink GR, Marshall JC. Mechanisms of hemispheric specialization: insights from analyses of connectivity. Neuropsychologia. 2007;45(2):209–28. doi: 10.1016/j.neuropsychologia.2006.07.002 16949111 10.1016/j.neuropsychologia.2006.07.002 PMC2638113

[80] Kell CA, Keller C. Contributions of auditory, cognitive, and motor computations to lateralization of speech processing In: van Lieshout P, Maasen B, Terband H, editors. Speech Motor Control in Normal and Disordered Speech Future Developments in Theory and Methodology: American Speech-Language-Hearing Association; 2016 p. 25–46.

