# Supplementary figures and images for: Functional hemispheric asymmetries during the planning and manual control of virtual avatar movements
Source: PLoS One. 2017 Sep 28;12(9):e0185152. doi: 10.1371/journal.pone.0185152 (PMC5619738; doi:10.1371/journal.pone.0185152)

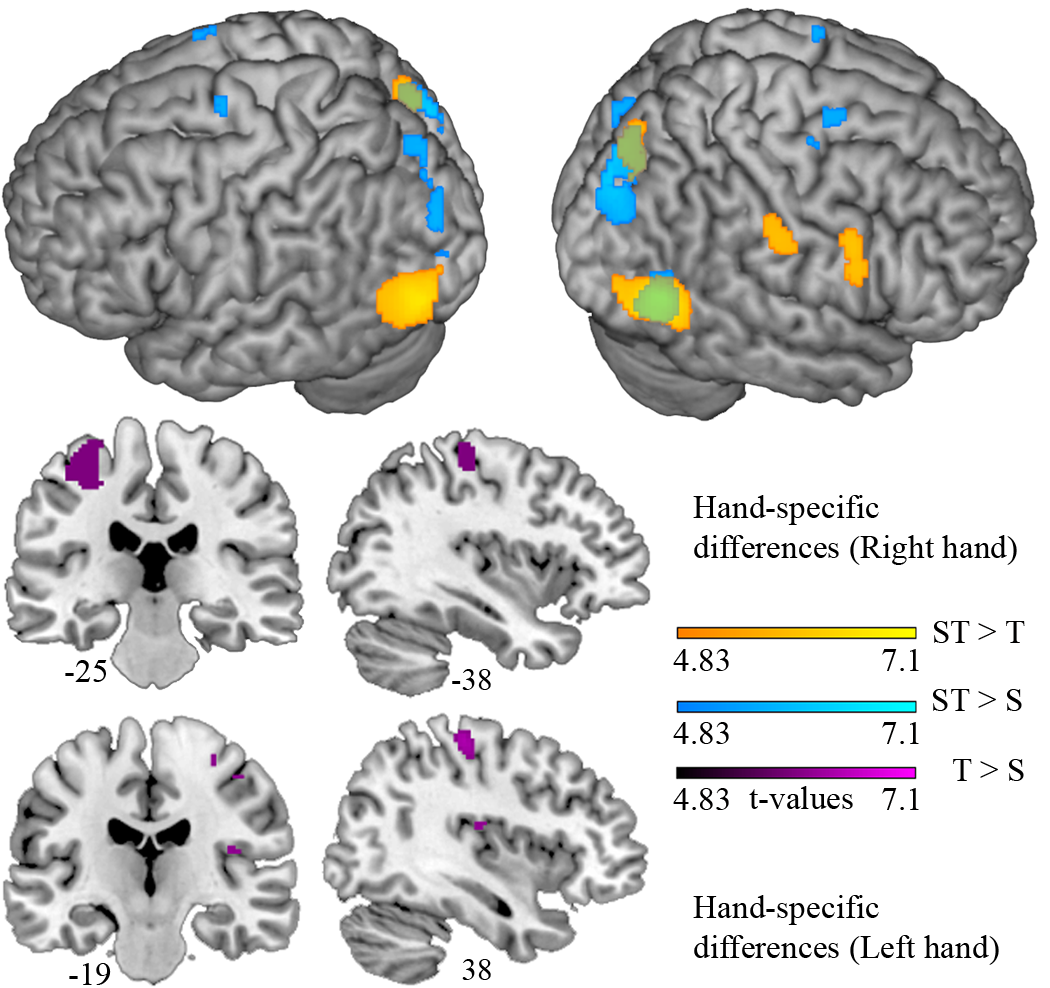

Supplement: S1 Fig — Upper row: Effector-independent differences between conditions during planning. Lower rows: Hand-specific differences between conditions during planning. Differences that were hand-nonspecific are masked out. Colors represent the different pairwise comparisons contrasts and their overlap (high spatial, high temporal processing demands (ST) in yellow; high spatial, low temporal processing demands (S) in blue; low spatial high temporal processing demands (T) in purple; overlay ST ∩ S in green). SPMs are overlaid on a representative brain normalized to MNI space (pFWE < 0.05 on the voxel level). (TIF) [file pone.0185152.s002.tif]
