# Supplementary material for: Functional hemispheric asymmetries during the planning and manual control of virtual avatar movements
Source: PLoS One. 2017 Sep 28;12(9):e0185152. doi: 10.1371/journal.pone.0185152 (PMC5619738; doi:10.1371/journal.pone.0185152)
Supplement: S1 Table — (DOCX) [file pone.0185152.s003.docx]

**S1 Table. Results of the conjunction analysis over all conditions and hand-dependent BOLD signal differences averaged over the three conditions in the combined model**

| **Anatomical region** | **BA^a^** | **k^b^** | **L/R** | **x** | **y** | **z** | ***t*** | **k^b^** | **L/R** | **x** | **y** | **z** | ***t*** | **k^b^** | **L/R** | **x** | **y** | **z** | ***t*** |
| --- | --- | --- | --- | --- | --- | --- | --- | --- | --- | --- | --- | --- | --- | --- | --- | --- | --- | --- | --- |
|  |  |  |  |  |  |  |  |  | **Left hand > right hand** | | | | |  | **Right hand > left hand** | | | | |
| Intraparietal sulcus (IPS) | 40 | 161 | R | 46 | -46 | 52 | 7.84 |  | - | - | - | - | - |  | - | - | - | - | - |
| Inferior parietal lobule (IPL) | 40 |  | R | 50 | -40 | 56 | 8.21 |  | - | - | - | - | - |  | - | - | - | - | - |
| Inferior parietal lobule (IPL) | 40 |  | R | 56 | -36 | 50 | 7.61 |  | - | - | - | - | - |  | - | - | - | - | - |
| Supramarginal gyrus (SMG) | 40 | 13 | L | -62 | -40 | 32 | 6.46 |  | - | - | - | - | - |  | - | - | - | - | - |
| Supramarginal gyrus (OP1/SII) | 40 | 36 | L | -66 | -22 | 18 | 7.93 |  | - | - | - | - | - |  | - | - | - | - | - |
| Supramarginal gyrus (OP1/SII) | 40 |  | L | -62 | -30 | 16 | 7.92 |  | - | - | - | - | - |  | - | - | - | - | - |
| Temporal pole | 38 | 64 | L | -58 | 14 | -2 | 6.57 |  | - | - | - | - | - |  | - | - | - | - | - |
| Temporal pole | 38 | 36 | R | 58 | 12 | -8 | 6.6 |  | - | - | - | - | - |  | - | - | - | - | - |
| Thalamus |  | 26 | L | -16 | -12 | 16 | 8.78 |  | - | - | - | - | - |  | - | - | - | - | - |
| Putamen |  | 18 | R | 32 | -20 | 0 | 5.67 |  | - | - | - | - | - | 55 | L | -30 | -14 | -4 | 6.87 |
| Caudate nucleus |  | 53 | R | 18 | -8 | 18 | 10.1 |  | - | - | - | - | - |  | - | - | - | - | - |
| Caudate nucleus |  |  | R | 22 | -22 | 14 | 6.3 |  | - | - | - | - | - |  | - | - | - | - | - |
| Cerebellar vermis |  | 35 |  | 2 | -52 | -2 | 7.41 |  | - | - | - | - | - |  | - | - | - | - | - |
| Cerebellum (Lobule V) |  |  | - | - | - | - | - | 5 | L | -8 | -50 | -10 | 6.8 | 7 | R | 8 | -48 | -18 | 7.45 |
| Cerebellum (Lobule V) |  |  | - | - | - | - | - | 9 | L | -24 | -40 | -28 | 8.16 |  | - | - | - | - | - |
| Cerebellum (Lobule VI) |  | 123 | L | -34 | -56 | -30 | 11.4 |  | - | - | - | - | - |  | - | - | - | - | - |
| Cerebellum (Lobule VI) |  |  | L | -24 | -66 | -26 | 9.3 |  | - | - | - | - | - | 5 | R | 14 | -56 | -24 | 9 |

Reported local maxima are significant with *p*_FWE_ < 0.05 at the voxel level.

BA = Brodmann’s area, k = cluster size, L/R = left hemisphere/right hemisphere, OP1 = dorsal posterior parietal opercular area , SII = secondary somatosensory cortex
