# Supplementary material for: Functional hemispheric asymmetries during the planning and manual control of virtual avatar movements
Source: PLoS One. 2017 Sep 28;12(9):e0185152. doi: 10.1371/journal.pone.0185152 (PMC5619738; doi:10.1371/journal.pone.0185152)
Supplement: S2 Table — (DOCX) [file pone.0185152.s004.docx]

**S2 Table. Brain regions exhibiting hand-specific lateralized BOLD responses**

| **Anatomical region** | |  | **k** | **L/R** | | **x** | | **y** | | **z** | | **t** | | **k** | **L/R** | | **x** | | **y** | | **z** | | **t** | |
| --- | --- | --- | --- | --- | --- | --- | --- | --- | --- | --- | --- | --- | --- | --- | --- | --- | --- | --- | --- | --- | --- | --- | --- | --- |
|  |  | |  | **Lateralization left hand** | | | | | | | | |  | | **Lateralization right hand** | | | | | | | | |  |
| **Activated during planning** | | | | | | | | | | | | | | | | | | | | | | | |  |
| Lingual gyrus (V3/V4) | **ST** | | 314 | R | 20 | | -72 | | -12 | | 7.43 | | 373 | | L | -20 | | -80 | | -12 | | 8.05 | |  |
|  | **S** | | 97 | R | 16 | | -76 | | -12 | | 6.35 | | 283 | | L | -44 | | -76 | | -6 | | 6.50 | |  |
|  | **T** | | 325 | R | 22 | | -70 | | -10 | | 6.49 | | 100 | | L | -18 | | -82 | | -12 | | 6.59 | |  |
| Pre/Postcentral gyrus (M1/S1) | **ST** | | 1414 | R | 36 | | -24 | | 54 | | 14.56 | | 1354 | | L | -38 | | -26 | | 56 | | 12.74 | |  |
|  | **ST** | |  | - | - | | - | | - | | - | |  | | L | -22 | | -18 | | 68 | | 7.33 | |  |
|  | **S** | | 1201 | R | 36 | | -24 | | 54 | | 12.02 | | 1286 | | L | -36 | | -24 | | 56 | | 11.19 | |  |
|  | **S** | |  | R | 52 | | -22 | | 52 | | 8.06 | |  | | L | -26 | | -20 | | 74 | | 7.41 | |  |
|  | **T** | | 1598 | R | 34 | | -24 | | 56 | | 18.14 | | 2126 | | L | -34 | | -26 | | 54 | | 18.44 | |  |
|  | **T** | |  | - | - | | - | | - | | - | |  | | L | -24 | | -12 | | 68 | | 8.56 | |  |
| Rolandic Operculum | **ST** | | 40 | R | 42 | | -20 | | 14 | | 6.65 | | 11 | | L | -40 | | -18 | | 16 | | 5.33 | |  |
|  | **S** | |  | - | - | | - | | - | | - | | 12 | | L | -42 | | -16 | | 18 | | 5.51 | |  |
|  | **T** | | 119 | R | 38 | | -20 | | 16 | | 8.24 | | 219 | | L | -38 | | -18 | | 16 | | 8.63 | |  |
| CMA | **ST** | | 42 | R | 6 | | -16 | | 50 | | 5.39 | |  | | - | - | | - | | - | | - | |  |
|  | **S** | | 32 | R | 6 | | -6 | | 48 | | 5.47 | |  | | - | - | | - | | - | | - | |  |
|  | **T** | | 186 | R | 8 | | -14 | | 50 | | 8.41 | | 75 | | L | -6 | | -10 | | 48 | | 6.35 | |  |
| Insula lobe | **ST** | |  | - | - | | - | | - | | - | | 45 | | L | -44 | | -4 | | -6 | | 6.69 | |  |
|  | **S** | | 1 | R | 38 | | 16 | | 6 | |  | | 64 | | L | -42 | | -4 | | 6 | | 6.49 | |  |
|  | **T** | |  | - | - | | - | | - | | - | | 155 | | L | -42 | | -4 | | 8 | | 7.51 | |  |
| Thalamus | **ST** | | 10 | R | 16 | | -22 | | 0 | | 6.06 | |  | | - | - | | - | | - | | - | |  |
|  | **S** | | 11 | R | 18 | | -22 | | 2 | | 5.64 | | 32 | | L | -16 | | -20 | | 4 | | 6.27 | |  |
|  | **T** | | 107 | R | 18 | | -20 | | 4 | | 8.64 | | 120 | | L | -16 | | -22 | | 4 | | 9.52 | |  |
| **Activated during planning and online control** | | | | | | | | | | | | | | | | | | | | | | | |  |
| Putamen | **ST** | | 20 | R | 30 | | -14 | | -2 | | 6.12 | |  | | - | - | | - | | - | | - | |  |
|  | **S** | | 5 | R | 32 | | -12 | | 0 | | 5.31 | |  | | - | - | | - | | - | | - | |  |
|  | **T** | | 30 | R | 30 | | -14 | | -2 | | 6.96 | | 4 | | L | -26 | | -22 | | 6 | | 5.17 | |  |
| Cerebellum (Lobule V) | **ST** | | 3 | L | -24 | | -40 | | -30 | | 5.12 | |  | | - | - | | - | | - | | - | |  |
|  | **S** | | 2 | L | -22 | | -40 | | -28 | | 5.01 | |  | | - | - | | - | | - | | - | |  |
|  | **T** | | 11 | L | -22 | | -40 | | -28 | | 8.54 | |  | | - | - | | - | | - | | - | |  |
|  | **T** | | 6 | L | -8 | | -50 | | -10 | | 7.12 | | 10 | | R | 8 | | -48 | | -18 | | 7.83 | |  |
| Cerebellum (Lobule VI) | **ST** | |  | - | - | | - | | - | | - | | 1 | | R | 14 | | -56 | | -24 | | 5.04 | |  |
|  | **S** | |  | - | - | | - | | - | | - | |  | | - | - | | - | | - | | - | |  |
|  | **T** | |  | - | - | | - | | - | | - | | 5 | | R | 14 | | -56 | | -24 | | 8.13 | |  |

Reported local maxima are significant with *p*_FWE_ < 0.05 at the voxel level. Only the two highest local maxima per cluster are displayed.

k = cluster size, L/R = left hemisphere/right hemisphere, M1 = primary motor cortex, S1 = primary somatosensory cortex, V3 = visual area 3, V4 = visual area 4
