# Supplementary material for: Functional hemispheric asymmetries during the planning and manual control of virtual avatar movements
Source: PLoS One. 2017 Sep 28;12(9):e0185152. doi: 10.1371/journal.pone.0185152 (PMC5619738; doi:10.1371/journal.pone.0185152)
Supplement: S3 Table — (DOCX) [file pone.0185152.s005.docx]

**S3 Table. Brain regions exhibiting different BOLD responses depending on condition**

| **Anatomical region** | **BA** | **k** | **L/R** | **x** | **y** | **z** | ***t*** | **k** | **L/R** | **x** | **y** | **z** | ***t*** | **k** | **L/R** | **x** | **y** | **z** | ***t*** | **k** | **L/R** | **x** | **y** | **z** | ***t*** |
| --- | --- | --- | --- | --- | --- | --- | --- | --- | --- | --- | --- | --- | --- | --- | --- | --- | --- | --- | --- | --- | --- | --- | --- | --- | --- |
|  |  |  |  | **ST** | **>** | **T** |  |  |  | **ST** | > | S |  |  |  | **S** | **>** | **T** |  |  |  | **T** | **>** | **S** |  |
| **Occipital and Temporal areas** |  |  |  |  |  |  |  |  |  |  |  |  |  |  |  |  |  |  |  |  |  |  |  |  |  |
| Calcarine gyrus (V1) | 17 | 41 | L | -6 | -76 | 12 | 6.7 | 2096 | L | -6 | -78 | -4 | 14 |  | - | - | - | - | - | - | - | - | - | - | - |
| Lingual gyrus (V1) | 17 |  | - | - | - | - | - |  | L | -2 | -78 | 4 | 13 |  | - | - | - | - | - | 1726 | L | -4 | -76 | -8 | 9.7 |
| Lingual gyrus (V2) | 18 |  | - | - | - | - | - |  | L | -10 | -66 | -2 | 12 |  | - | - | - | - | - |  | - | - | - | - | - |
| Lingual gyrus (V3) | 18 | 6 | L | -14 | -62 | -4 | 5.7 | - | - | - | - | - | - |  | - | - | - | - | - |  | R | 20 | -68 | -8 | 9.9 |
| Lingual gyrus (V4) | 18 | - | - | - | - | - | - | - | - | - | - | - | - |  | - | - | - | - | - |  | L | -18 | -70 | -10 | 9.3 |
| Superior occipital gyrus | 19 | - | - | - | - | - | - | 19 | L | -12 | -82 | 44 | 5.8 |  | - | - | - | - | - | - | - | - | - | - | - |
| Superior occipital gyrus | 19 | - | - | - | - | - | - | 430 | R | 20 | -84 | 38 | 8.9 |  | - | - | - | - | - | 25 | R | 24 | -78 | 24 | 5.6 |
| Superior occipital gyrus | 19 | - | - | - | - | - | - |  | R | 24 | -80 | 30 | 8.2 |  | - | - | - | - | - |  | - | - | - | - | - |
| Middle occipital gyrus | 19 | - | - | - | - | - | - | 79 | L | -22 | -86 | 20 | 6.4 |  | - | - | - | - | - | - | - | - | - | - | - |
| Middle occipital gyrus (LOC) | 19 | 446 | L | -46 | -80 | -2 | 9.1 | - | - | - | - | - | - | 206 | L | -46 | -80 | -2 | 6.8 | - | - | - | - | - | - |
| Middle occipital gyrus (LOC) | 19 |  | - | - | - | - | - | - | - | - | - | - | - |  | L | -38 | -84 | -4 | 6.44 | - | - | - | - | - | - |
| Inferior occipital gyrus (LOC) | 19/37 |  | - | - | - | - | - | - | - | - | - | - | - |  | L | -44 | -66 | -4 | 5.18 | **-** | - | - | - | - | - |
| Inferior occipital gyrus (LOC) | 19/37 | 693 | R | 42 | -80 | -2 | 6.4 |  | - | - | - | - | - |  | - | - | - | - | - | - | - | - | - | - | - |
| Middle temporal gyrus (V5/MT) | 37 | 693 | R | 46 | -66 | 0 | 11 | 249 | R | 46 | -64 | 2 | 6.6 | 181 | R | 44 | -46 | -2 | 7.15 | - | - | - | - | - | - |
| Middle temporal gyrus (LOC) | 37 |  | - | - | - | - | - |  | R | 52 | -66 | 10 | 5.9 |  | - | - | - | - | - | - | - | - | - | - | - |
| Middle temporal gyrus (LOC) | 37 |  | - | - | - | - | - |  | R | 52 | -72 | -2 | 5.7 |  | - | - | - | - | - | - | - | - | - | - | - |
| Fusiform gyrus | 37 | 3 | R | 40 | -48 | -16 | 5.3 | - | - | - | - | - | - | - | - | - | - | - | - | - | - | - | - | - | - |
| **Parietal areas** |  |  |  |  |  |  |  |  |  |  |  |  |  |  |  |  |  |  |  |  |  |  |  |  |  |
| Superior parietal lobule | 7 | 94 | R | 14 | -47 | 52 | 5.6 | - | - | - | - | - | - | - | - | - | - | - | - | - | - | - | - | - | - |
| Supramarginal gyrus (SMG) | 40 | 71 | R | 60 | -22 | 36 | 5.7 | - | - | - | - | - | - | - | - | - | - | - | - | - | - | - | - | - | - |
| Supramarginal gyrus (SMG) | 40 |  | - | - | - | - | - | - | - | - | - | - | - | - | - | - | - | - | - | - | - | - | - | - | - |
| **Frontal areas** |  |  |  |  |  |  |  |  |  |  |  |  |  |  |  |  |  |  |  |  |  |  |  |  |  |
| Superior frontal gyrus (PMd) | 6 | - | - | - | - | - | - | 12 | R | 28 | -10 | 54 | 5.4 | - | - | - | - | - | - | - | - | - | - | - | - |
| Superior frontal gyrus (PMd) | 6 | - | - | - | - | - | - | 16 | R | 26 | -2 | 68 | 5.3 | - | - | - | - | - | - | - | - | - | - | - | - |
| Superior frontal gyrus (PMd) | 6 | - | - | - | - | - | - | 7 | L | -24 | -8 | 72 | 5.4 | - | - | - | - | - | - | - | - | - | - | - | - |
| Precentral gyrus (PMv) | 6 | 111 | R | 58 | 6 | 32 | 6.1 | - | - | - | - | - | - | - | - | - | - | - | - | - | - | - | - | - | - |
| Inferior frontal gyrus (PMv) | 44 |  | R | 52 | 6 | 18 | 6.8 | - | - | - | - | - | - | - | - | - | - | - | - | - | - | - | - | - | - |

Reported local maxima are significant with p_FWE_ < 0.05 at the voxel level.

k = cluster size, L/R = left hemisphere/right hemisphere, LOC = lateral occipital cortex, PMd = premotor cortex dorsal, PMv = premotor cortex ventral, V1/V2/V3/V4 = visual area V1/2/3/4, V5/MT = visual area V5/middle temporal area
